# Understanding Vietnamese chicken farmers’ knowledge and practices related to antimicrobial resistance using an item response theory approach

**DOI:** 10.3389/fvets.2024.1319933

**Published:** 2024-04-05

**Authors:** Sandra Nohrborg, Thinh Nguyen-Thi, Huyen Nguyen Xuan, Johanna Lindahl, Sofia Boqvist, Josef D. Järhult, Ulf Magnusson

**Affiliations:** ^1^Department of Clinical Sciences, Swedish University of Agricultural Sciences, Uppsala, Sweden; ^2^International Livestock Research Institute, Regional Office for East and Southeast Asia, Hanoi, Vietnam; ^3^Department of Bacteriology, National Institute of Veterinary Research, Hanoi, Vietnam; ^4^Department of Animal Health and Antimicrobial Strategies, National Veterinary Institute, Uppsala, Sweden; ^5^Department of Biomedical Sciences and Veterinary Public Health, Swedish University of Agricultural Sciences, Uppsala, Sweden; ^6^Department of Medical Sciences, Zoonosis Science Center, Uppsala University, Uppsala, Sweden

**Keywords:** antimicrobial resistance, antimicrobial use, item response theory, chicken farming, poultry, Vietnam, farmers knowledge and practice

## Abstract

**Introduction:**

Antimicrobial resistance (AMR) poses a threat to animal and human health, as well as food security and nutrition. Development of AMR is accelerated by over- and misuse of antimicrobials as seen in many livestock systems, including poultry production. In Vietnam, high AMR levels have been reported previously within poultry production, a sector which is dominated by small-scale farming, even though it is intensifying. This study focuses on understanding small- and medium-scale chicken farmers’ knowledge and practices related to AMR by applying an item response theory (IRT) approach, which has several advantages over simpler statistical methods.

**Methods:**

Farmers representing 305 farms in Thai Nguyen province were interviewed from November 2021 to January 2022, using a structured questionnaire. Results generated with IRT were used in regression models to find associations between farm characteristics, and knowledge and practice levels.

**Results:**

Descriptive results showed that almost all farmers could buy veterinary drugs without prescription in the local community, that only one third of the farmers received veterinary professional advice or services, and that the majority of farmers gave antibiotics as a disease preventive measure. Regression analysis showed that multiple farm characteristics were significantly associated to farmers’ knowledge and practice scores.

**Conclusion:**

The study highlights the complexity when tailoring interventions to move towards more medically rational antibiotic use at farms in a setting with high access to over-the-counter veterinary drugs and low access to veterinary services, since many on-farm factors relevant for the specific context need to be considered.

## 1 Introduction

It is widely acknowledged that antimicrobial use (AMU) is closely linked to the development of antimicrobial resistance (AMR), a mechanism in a microbe to survive exposure to an antimicrobial it initially was sensitive to (1, 2). Even though AMR is a naturally occurring phenomenon in bacteria, the development is accelerated by over- and misuse of antibiotics as seen in many livestock production systems and health care facilities. Besides being used, as intended, to treat disease, antibiotics might for example also be used for disease prevention and/or growth promotion (2, 3). The obvious consequence of AMR is treatment failure, which negatively impacts animal welfare, reduces animals’ growth and productivity, and increases mortality rates (4). Subsequently, this has serious effects since hundreds of millions of people around the world depend on livestock for their livelihoods, as well as for their food security and nutrition, especially in low- and middle-income countries (LMICs) (5).

Globally, the use of antibiotics in the livestock sector is extensive, estimated to exceed the use in the human sector, and concerning levels of AMR have been documented (6, 7). Further, as resistant bacteria can spread between animals and humans, directly or indirectly, AMU in the livestock sector also poses a threat to public health (8–10). Therefore, serious effort needs to be put into reducing over- and misuse in animal production for the sake of both human and animal health.

Southeast Asia has a rapidly intensifying livestock sector, mainly in the monogastric animal production, i.e., poultry and pig production (11). More animals and higher animal densities in farms, often in combination with insufficient disease preventive measures, lead to higher disease pressure and subsequent increased antibiotic use (ABU), including prophylactic use (6, 12, 13). The connections between intensified animal rearing and increased ABU have contributed to Southeast Asia becoming one of the hotspots for AMR emergence (7, 14). It has further been shown that, since the beginning of the millennium, AMR levels in LMICs have increased the most within poultry and pig production which is consistent with the intensification of those sectors (7).

Apart from increased disease pressure as the livestock sector is intensified, extensive and inappropriate ABU might be further facilitated by weak legislation and guidelines on antibiotic sales and use, and in some settings insufficient enforcement of such regulations (13, 15, 16). For example, in Vietnam, where regulations state that using antibiotics from growth promotion is prohibited (17), over-the-counter sales of antibiotics without prescription are still common, as in many LMICs, which makes implementation of the regulations difficult (13, 18).

As on the global level, the amount of antibiotics used in livestock production in Vietnam exceeds that in humans, with an estimation that >70% of the total amount of antibiotics used in the country in 2015 was used in livestock (19). Further, several studies have reported use of antibiotics critically important to human health, as classified by the World Health Organization (WHO), in Vietnamese farms (12, 20–23).

However, inappropriate use of antibiotics cannot solely be explained by intensified livestock production and weakness in regulatory systems. Different factors at both farmer and community level may affect the use of antibiotics, such as economic incentives, lack of knowledge, and access to animal health services and veterinary drug shops (16, 24). Further, it should be acknowledged that the drivers on different levels might vary across local socio-economic contexts within the same regulatory framework (25). To be able to apply a tailored bottom-up approach to reduce ABU at farm level, there is a need to understand those context-specific drivers among farmers (14, 26). Therefore, questionnaire-based knowledge, attitude and practice studies (KAPs) have become common and valuable research tools (13, 15, 25, 27–30).

Most published farmer KAP studies are based on classical test theory (CTT), which has several limitations, like the equal value of the questions in the test, and difficulties in knowing that the test actually measures the trait of interest due to the lack of an underlying scale. Therefore, another psychometric method called item response theory (IRT) was chosen for this study. In IRT, the foundation is the relationship between a person’s unobservable measurement of the underlying trait, e.g., knowledge about AMR, and the probability of different responses to the items in the test. Further, the scores of respondents are measured on a standardized scale and based on the individual difficulty and quality of the questions (or items) in the test. IRT has recently started to transition into the field of veterinary and public health research (29, 31–33). The current study is one of the first to use IRT for evaluating farmers’ practice ability and knowledge regarding ABU and AMR development, and to the authors’ knowledge, the first in the Southeast Asia region and in poultry production.

The main objectives of the study were to: (1) identify which demographic factors that affect farmers’ AMR-related practices and knowledge in small- and medium-scale chicken farms in Vietnam, (2) describe farmers’ access to, and use of, veterinary pharmaceuticals and animal health services, and (3) to assess the feasibility of IRT as a method when performing AMR-related KAP studies.

## 2 Materials and methods

### 2.1 Study area

Small-scale farming is the most common type of livestock production in Vietnam. However, a trend of increasing farm sizes is seen for several species, including chicken. Even though the number of larger farms is increasing, raising more than 100 chickens is still uncommon, with around 6% of farms being that large in 2020, and 50% of farms had only 20–49 chickens (34).

**Figure 1:**
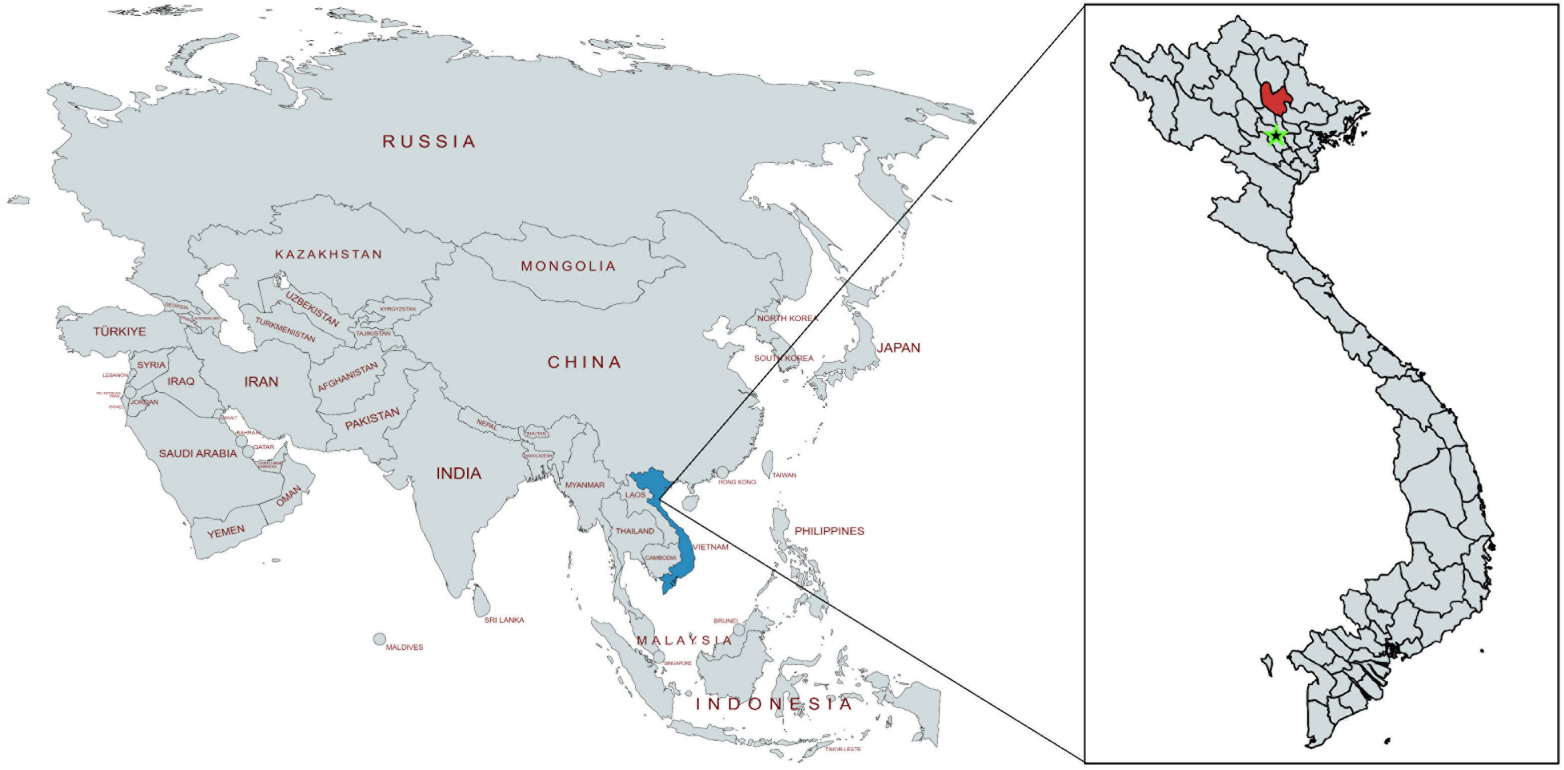
Map of the continent of Asia (left) with Vietnam marked in blue, and map of Vietnam (right) with the province Thai Nguyen marked in red. Star marks the capital of Hanoi for reference. Source: https://mapchart.net, accessed September 29, 2023, license: https://creativecommons.org/licenses/by-sa/4.0/.

The study was conducted in Thai Nguyen province which is located in the northern midlands and mountain areas north of the capital of Hanoi (see Figure 1), with a human population of 1.3 million people in 2022 (35). In 2020, 49% of all rural households in the province were engaged in agriculture, forestry or fishery (34), and the number of households that kept chicken in 2016 was approximately 173 thousand, corresponding to about 2% of the chicken raising households in the country (36). The estimated poultry population of the province was around 14 million in 2022, accounting for 2.6% of the number of poultry in the country (35). Thai Nguyen province was selected based on its large chicken population, the distribution of chicken farms between districts within the province, and its proximity to the capital Hanoi.

Farms from three districts in the province were included in the study: Thai Nguyen City, Dong Hy and Vo Nhai, with 360.0, 94.7 and 69.8 thousand inhabitants, respectively, in 2022 (37). Of the three districts, Vo Nhai has the largest land area and Thai Nguyen City the smallest. In Thai Nguyen City, the majority of the population resides in urban areas while in Dong Hy and Vo Nhai, the rural population is in majority (38). The district selection was based on the districts’ different chicken population sizes (Thai Nguyen City having the most and Vo Nhai the least) and profiles (Thai Nguyen City being more urban and Dong Hy and Vo Nhai more rural) in order for the districts to be as representative as possible for the province as a whole.

### 2.2 Study population

The chicken farms in the three selected districts of Thai Nguyen province were categorized as small scale, with 20–49 chickens, and medium scale, with 100–499 chickens. These farm-size categories made up 46.2 and 5.3%, for small- and medium-scale farms respectively, of the total number of chicken farms in the province in 2016 (36).

### 2.3 Sampling design

A sample size of 300 farms in total was considered sufficient for the purpose of the study, and was distributed evenly between the two farm-size categories. To select farms to be included, lists of all small- and medium-scale chicken-raising households in each district, according to the above-mentioned definitions, were collected from the sub-Department of Animal Health (sub-DAH) in Thai Nguyen province. To avoid an over- or under-representation of a district, the numbers of farms to be included were stratified according to the proportion of farms in each farm size category in each district. After rounding, the number of small- and medium-scale farms from each district were therefore distributed as follows: Thai Nguyen City, 8 and 42; Dong Hy, 24 and 32; and Vo Nhai, 119 and 77, resulting in a sample size of 302 farms.

Two sampling frames, one for each farm-size category, were created from the obtained lists of chicken-raising households for each district. For logistic reasons, villages with less than five households were removed from the sampling frames. According to the stratification, households were then randomly selected via an online randomization tool (39). If one household needed to be replaced for any reason, a nearby farm that met the requirements was included instead. The main reasons for household replacement were: the farmer being busy or not giving consent to participate, or having too many, too few or no chicken at the time of visit. In total, 159 households were replaced (53%).

### 2.4 Data collection

To investigate the farmers’ practices and knowledge regarding antibiotics and AMR, a structured questionnaire of 102 questions was developed and divided into the following sections: (a) General information and farm location, (b) Farm characteristics, general management routines and access to animal health services and veterinary drugs, (c) Chicken disease issues, (d) Disease prevention, disease management and treatment routines, and (e) Knowledge about antibiotic use and AMR (see Supplementary material S1).

The questionnaire was initially developed in English and then translated into Vietnamese. To discover possible misinterpretations, back-translation was performed by a person outside of the research team. The survey was conducted in interview format by five trained enumerators from the National Institute for Veterinary Research (NIVR), Hanoi. Answers were recorded on tablets through the online survey tool platform Netigate (40).

The enumerators participated in a two-day training where they got familiar with the survey tool and the questionnaire. They also got to practice interviewing with each other and to perform pilot interviews in the field, on chicken farms not situated in the study area. Feedback on the questionnaire from the training and field test was taken into account and changes to the questionnaire were made accordingly. The survey was conducted from the 25 November 2021 to the 20 January 2022.

### 2.5 Statistical methods

#### 2.5.1 Data processing

The questionnaire data was downloaded from Netigate to Microsoft Excel where the dataset was processed. Issues such as duplicate farms, where the same ID number had been erroneously typed for two farms or where the same farm had been entered twice into Netigate, were resolved. The corrected dataset consisted of responses from 305 farms: 51 in Thai Nguyen City, 56 in Dong Hy and 198 in Vo Nhai.

Additional data cleaning was performed, and free text answers were translated from Vietnamese to English and added to the dataset. The dataset was then imported to the statistical software STATA (41) for further data processing and statistical analyses.

Descriptive statistics were compiled for all items (questions) in the questionnaire. Practice and knowledge items were further evaluated according to an IRT workflow (see Section 2.5.2.2.), and items that fit the criteria were included in IRT scales, two for practices (Practice 1, relating to disease management and antibiotic treatment; and Practice 2, relating to disease prevention) and one for knowledge (See Supplementary Table S9). The mean theta values generated through the IRT analyses for groups in different variables of interest (see Section 2.5.2.4.) were compared using one-way ANOVA, and the effects of the variables of interest on theta were evaluated through multilevel mixed-effects linear regression. A 5% significance level was used for all statistical analyses.

#### 2.5.2 Item response theory

##### 2.5.2.1 Concept

When using IRT, the underlying trait of interest, e.g., ability or knowledge, is measured in theta (θ), which is standardized with a mean value of 0 and a standard deviation of 1, i.e., a person with a theta value of 0 has an average level of ability or knowledge. A theta value >0 reflects a higher than average ability or knowledge, and correspondingly, a theta value of <0 reflects a lower than average ability or knowledge.

The IRT model used in this study was a two-parameter logistic (2pl) model. The 2pl model takes into account two parameters of each item in the scale, discrimination and difficulty (42). The discrimination of an item is the measure of how good it can separate people above and below a certain theta level, i.e., how much information the item contributes with to explain a person’s ability or knowledge. As for the underlying trait, the difficulty is also measured in theta. A person with the same theta score as the difficulty of the question has a 50% probability of answering the item correctly or desirably.

##### 2.5.2.2 Workflow and scale generation

Binary variables were created from all practice and knowledge questions (38 practice and 18 knowledge questions). Options in practice questions were coded as desirable or undesirable, and knowledge questions as correct or incorrect, from an AMR mitigation, development and spread perspective (see Tables 1, 2). Five practice questions were removed from the scale generation due to too few responses, and one where all respondents answered undesirably, thus not providing any information regarding differences in ability/practice. As a result, 32 practice questions were left.

**Table 1:** Antimicrobial resistance (AMR) related practices among small- and medium-scale chicken farmers in Thai Nguyen City, Dong Hy and Vo Nhai districts in Vietnam.

Item	Option category	Option	% (number)
Do other animals at the farm have access to the areas where your chickens/hens are kept?^b^ (*n* = 305)	Desirable	No, There are no other animal species at my farm	31.8 (97)
	Undesirable	Yes	68.2 (208)
Do your chickens/hens mix with animals from outside your own farm?^b^ (*n* = 305)	Desirable	Rarely, Never	89.2 (272)
	Undesirable	Yes, often, Yes, sometimes	10.8 (33)
If you slaughter chickens/hens at the farm, do you have a specific area for slaughter that is separated from live animals?^d^ (*n* = 129)	Desirable	Yes	85.3 (110)
	Undesirable	No	14.7 (19)
How do you usually handle manure from your chickens/hens?^b^^,^^c^ (*n* = 305)	Desirable	Use or sell/give after treatment of the manure, Use or sell/give after at least 1 month of composting, Use for fuel (incl. biogas)	44.6 (136)
	Undesirable	Do nothing, Discard into the environment, Use or sell/give untreated as fertilizer	53.8 (164)
What do you usually do with chickens/hens that die from disease?^b^^,^^c^ (*n* = 305)	Desirable	Burn/destruct, Bury in the ground	78.4 (239)
	Undesirable	Throw in the trash, Use as animal feed, Use for household consumption, Sell at local market, Sell to other farmers as animal feed, Leave on the ground	19.7 (60)
Do you usually empty the animal houses/areas between batches of chickens/hens (all-in/all-out system)? (*n* = 305)	Desirable	Yes	15.1 (46)
	Undesirable	No	84.9 (259)
If you use all-in/all-out system, do you remove litter, manure and clean/disinfect animal houses before next batch?^d^ (*n* = 46)	Desirable	Yes	93.5 (43)
	Undesirable	No	6.5 (3)
If you do not use an all-in/all-out system, how often do you remove litter, manure and clean/disinfect animal houses/areas where the chickens/hens are kept?^b^ (*n* = 259)	Desirable	Once a week or more often, Every second week, Once a month	65.6 (170)
	Undesirable	More seldom	34.4 (89)
If you have access to buying pharmaceuticals/veterinary drugs, where do you most commonly buy them for your chickens/hens?^a^^,^^c^ (*n* = 294)	Desirable	Via a governmental veterinarian, directly or via prescription, Via a private veterinarian, directly or via prescription	6.5 (19)
	Undesirable	From other farmers, At markets, At veterinary drug shop without prior prescription, From pharmaceutical company, From a feed provider	92.5 (272)
If you have access to animal health services, do you use them for treatment of disease among your chickens/hens and/or advice on disease prevention?^d^ (*n* = 97)	Desirable	Yes, mostly, Sometimes	87.6 (85)
	Undesirable	No	12.4 (12)
If you use animal health services for treatment and advice, which animal health service provider do you most commonly use?^c^^,^^d^ (*n* = 85)	Desirable	Governmental veterinarian, Private veterinarian	42.4 (36)
	Undesirable	Veterinary drug shop worker (not veterinarian), Staff of drug company	56.5 (48)
If the animal health services include laboratory testing and/or autopsies, do you use these services?^d^ (*n* = 31)	Desirable	Yes, when needed	58.1 (18)
	Undesirable	Sometimes, Never	41.9 (13)
Do you keep records of disease and mortality among your chickens/hens? (*n* = 305)	Desirable	Yes	1.0 (3)
	Undesirable	No	99.0 (302)
To prevent your chickens/hens from becoming sick, do you: Fence them?^b^ (*n* = 305)	Desirable	Yes	63.0 (192)
	Undesirable	No	37.0 (113)
To prevent your chickens/hens from becoming sick, do you: Usually isolate/quarantine newly bought animals for some time^b^ (*n* = 305)	Desirable	Yes	57.4 (175)
	Undesirable	No	42.6 (130)
To prevent your chickens/hens from becoming sick, do you: Give them antibiotics (*n* = 305)	Desirable	No	34.8 (106)
	Undesirable	Yes	65.2 (199)
To prevent your chickens/hens from becoming sick, do you: Give them feed that is supplemented with antibiotics (*n* = 305)	Desirable	No	67.9 (207)
	Undesirable	Yes	32.1 (98)
To prevent your chickens/hens from becoming sick, do you: Vaccinate (*n* = 305)	Desirable	Yes	54.1 (165)
	Undesirable	No	45.9 (140)
Do you: Wash your hands before entering the areas where your animals are kept? (*n* = 305)	Desirable	Yes	23.6 (72)
	Undesirable	No	76.4 (233)
Do you: Wash your hands after visiting the areas where your animals are kept? (*n* = 305)	Desirable	Yes	92.5 (282)
	Undesirable	No	7.5 (23)
Do you: Have separate footwear (e.g., gum boots) or plastic boot covers that you use only in the areas where your chickens/hens are kept?^b^ (*n* = 305)	Desirable	Yes	41.3 (126)
	Undesirable	No	58.7 (179)
Do you give your chickens/hens antibiotics to make them grow faster and/or better? (*n* = 305)	Desirable	No	98.0 (299)
	Undesirable	Yes	2.0 (6)
Do you give your hens antibiotics to make them lay more eggs? (*n* = 225)	Desirable	No	98.2 (221)
	Undesirable	Yes	0.9 (2)
Who will usually diagnose disease among the chickens/hens at the farm?^a^^,^^c^ (*n* = 305)	Desirable	Governmental veterinarian, Private veterinarian	13.4 (41)
	Undesirable	Myself, Veterinary drug shop worker (not veterinarian), Human doctor, Other farmer, Friend/family member	85.9 (262)
What do you usually do first when the chickens/hens at your farm get sick?^a^^,^^c^ (*n* = 305)	Desirable	Consult a governmental veterinarian, Consult a private veterinarian	40.0 (122)
	Undesirable	Nothing, Give them medicine(s) from a veterinary drug shop/market, Give them traditional medicine/vitamins/herbs, Give them medicine(s) that was left by a veterinarian at a previous visit	55.1 (168)
Do you usually isolate chickens/hens that become sick from the rest of the poultry in the flock?^b^ (*n* = 305)	Desirable	Yes	80.0 (244)
	Undesirable	No	20.0 (61)
When you use antibiotics to treat disease among your chickens/hens, which animals do you usually treat?^c^ (*n* = 305)	Desirable	Only the chickens/hens that are sick, All chickens/hens that are sick and all animals in contact with the sick chickens/hens	43.0 (131)
	Undesirable	All poultry at the farm, All chickens/hens at the farm, All animals at the farm	51.5 (157)
From where do you usually get advice on when to use antibiotics for your chickens/hens?^a^^,^^c^ (*n* = 305)	Desirable	From a governmental veterinarian, From a private veterinarian	21.6 (66)
	Undesirable	I do not get advice, I use my own judgment, From other farmers, From veterinary drug shop worker (not veterinarian), From package/label of the medicine, From market sales person, From human doctor, From feed provider, From friends/family	72.8 (222)
When you use antibiotics to treat disease among your chickens/hens, for how long do you usually treat them?^a^^,^^c^ (*n* = 305)	Desirable	As advised by a governmental veterinarian, As advised by a private veterinarian	29.5 (90)
	Undesirable	Until animal(s) cured, Until animal(s) begin to recover, As advised by other (e.g., sales person, other farmer, family/friends, human doctor), As instructed on the package/label of the medicine, Until package is empty, One treatment only	64.3 (196)
When treating your chickens/hens with antibiotics, whose instructions do you usually follow on how to use them (dose, treatment length, administration route etc.)?^a^^,^^c^ (*n* = 305)	Desirable	A governmental veterinarian’s, A private veterinarian’s	23.9 (73)
	Undesirable	I do not get advice, I use my own judgment, Other farmers’, A veterinary drug shop worker’s (not veterinarian), The instructions on the package/label of the medicine, A market sales person’s, A human doctor’s, A feed provider’s, Friends’/family’s	69.8 (213)
When you use antibiotics to treat disease among chickens/hens, who usually administers the drug?^a^^,^^c^ (*n* = 305)	Desirable	Myself, after instructions from a governmental veterinarian, Myself, after instructions from a private veterinarian, Governmental veterinarian, Private veterinarian	33.4 (102)
	Undesirable	Myself, by own experience	60.0 (183)
Do you ever give a higher dose of antibiotics than the recommended to your chickens/hens?^c^ (*n* = 305)	Desirable	No	75.4 (230)
	Undesirable	Yes	19.3 (59)
Do you ever give a lower dose of antibiotics than the recommended to your chickens/hens?^c^ (*n* = 305)	Desirable	No	92.8 (283)
	Undesirable	Yes	1.3 (4)
Do you ever stop giving your chickens/hens antibiotics earlier than recommended if they seem healthy?^c^ (*n* = 305)	Desirable	No	79.7 (243)
	Undesirable	Yes	15.1 (46)
Does it happen that you give human medicines to your chickens/hens when they become sick? (*n* = 305)	Desirable	No	64.9 (198)
	Undesirable	Yes, often, Sometimes	35.1 (107)
If the antibiotic treatment of sick chickens/hens is not effective or does not work, what do you usually do?^a^^,^^c^ (*n* = 305)	Desirable	Contact governmental veterinarian, Contact private veterinarian, Euthanize the sick animal(s)	21.3 (65)
	Undesirable	Increase the dose, Switch to another antibiotic or combine the ongoing treatment with another antibiotic, Switch to other type of medicine, Switch to herbal/traditional medicine, Go back to the veterinary drug shop for advice (from non-veterinarian), Contact other person (not veterinarian) for advice, Slaughter the sick animal(s) for meat, Nothing	66.6 (203)
What do you usually do with expired/leftover veterinary antibiotics?^c^ (*n* = 305)	Desirable	Leave to pharmacy/veterinary drug shop	0 (0)
	Undesirable	Throw in the trash/latrine, Keep for later use, Give to other farmer	91.8 (280)
Do you keep records of the use of medicines for the chickens/hens at your farm (e.g., treatment dates, name of medicine, dose)? (*n* = 305)	Desirable	Yes	0.7 (2)
	Undesirable	No	99.3 (303)

Practices 1 = Ability to perform desirable practices related to disease management and treatment with antibiotics, Practices 2 = Ability to perform desirable practices regarding disease prevention.

a Item was included in Practices 1 scale.

b Item was included in Practices 2 scale.

c Item had options that could not be considered desirable or undesirable and these options were coded as missing but are still included in the number of responses.

d Item was removed from further analyses due to too few responses (details in Supplementary material S2).

**Table 2:** Knowledge about antibiotics and antimicrobial resistance (AMR) development and spread among small- and medium-scale chicken farmers in Thai Nguyen City, Dong Hy and Vo Nhai districts in Vietnam.

Item	Option category	Option	% (number)
What are antibiotics supposed to be used for? (*n* = 305)	Correct	Treat sick animals	68.9 (210)
	Incorrect	Prevent animals from becoming sick, Make animals grow faster/better, Prevent animals from becoming sick and make animals grow faster/better, Prevent animals from becoming sick and treat sick animals, Treat sick animals and make animals grow faster/better, Prevent animals from becoming sick, treat sick animals and to make animals grow faster/better	31.1 (95)
Antibiotics can treat all kinds of diseases (*n* = 305)	Correct	False	53.8 (164)
	Incorrect	True, Cannot answer	46.2 (141)
Antibiotics can treat diseases caused by viruses (*n* = 305)	Correct	False	50.8 (155)
	Incorrect	True, Cannot answer	49.2 (150)
Antibiotics can treat diseases caused by bacteria (*n* = 304)	Correct	True	87.2 (265)
	Incorrect	False, Cannot answer	12.8 (39)
Antibiotics are the same as anti-inflammatory drugs (*n* = 304)	Correct	False	38.8 (118)
	Incorrect	True, Cannot answer	61.2 (186)
Different types of antibiotics are needed for different diseases (*n* = 303)	Correct	True	80.2 (243)
	Incorrect	False, Cannot answer	19.8 (60)
As a general rule, you should stop treatment with antibiotics when the animal’s condition starts to improve (*n* = 303)	Correct	False	50.2 (152)
	Incorrect	True, Cannot answer	49.8 (151)
Using antibiotics too often can make diseases difficult to treat in the future^a^ (*n* = 304)	Correct	True	70.4 (214)
	Incorrect	False, Cannot answer	29.6 (90)
Animals can become resistant to antibiotics if antibiotics are used in the wrong way/too often (*n* = 304)	Correct	False	20.7 (63)
	Incorrect	True, Cannot answer	79.3 (241)
Bacteria that cause disease can become resistant to antibiotics if used in the wrong way/too often (*n* = 303)	Correct	True	72.9 (221)
	Incorrect	False, Cannot answer	27.1 (82)
Viruses that cause disease can become resistant to antibiotics if used in the wrong way/too often (*n* = 303)	Correct	False	32.3 (98)
	Incorrect	True, Cannot answer	67.7 (205)
Resistance against antibiotics can make it more difficult to succeed with antibiotic treatment in animals when they get sick^a^ (*n* = 303)	Correct	True	76.9 (233)
	Incorrect	False, Cannot answer	23.1 (70)
Bacteria resistant to antibiotics can spread from one animal to another^a^ (*n* = 304)	Correct	True	69.4 (211)
	Incorrect	False, Cannot answer	30.6 (93)
Bacteria resistant to antibiotics can spread between animals and humans^a^ (*n* = 302)	Correct	True	51.7 (156)
	Incorrect	False, Cannot answer	48.3 (146)
Bacteria resistant to antibiotics can spread from animals to humans through animal source foods, e.g., meat^a^ (*n* = 302)	Correct	True	45.0 (136)
	Incorrect	False, Cannot answer	55.0 (166)
Bacteria resistant to antibiotics can spread through manure from animals^a^ (*n* = 302)	Correct	True	52.0 (157)
	Incorrect	False, Cannot answer	48.0 (145)
Using too much antibiotics in animals can make it more difficult to treat some diseases in humans^a^ (*n* = 302)	Correct	True	54.3 (164)
	Incorrect	False, Cannot answer	45.7 (138)
Antibiotic resistance in human bacteria is only linked to the use of antibiotics in humans and not in animals (*n* = 299)	Correct	False	24.1 (72)
	Incorrect	True, Cannot answer	75.9 (227)

a The question was included in item response theory (IRT) model for knowledge.

The practice and knowledge questions were evaluated on the internal consistency reliability through Cronbach’s alpha (CA), a measure of whether the items in the scale consistently measures the same characteristic (43). A value of 0.7 or more for CA was considered sufficient.

In parallel with CA analyses, items were evaluated on other aspects reflecting the reliability and validity of the scales: positive/negative sign, item-rest correlation and item-total correlation. The average inter-item correlations for each scale were also generated.

Seventeen and five practice questions (for the Practices 1 and Practices 2 scale respectively; Supplementary Table S1), and four knowledge questions with negative signs were removed to make the scale plausible unidimensional (32). Thereafter, questions with an item-rest correlation <0.2 and item-total (sometimes called item-test) correlation <0.4 (42) were removed, seven for each practice scale and four for the knowledge scale. An average inter-item correlation of >0.2 was considered acceptable (44), which was fulfilled for all three scales.

To fit 2pl models, the assumption of unidimensionality needs to be fulfilled, i.e., there is a single underlying trait that accounts for the dependence among observations. Together with above mentioned evaluations, this assumption was tested through exploratory factor analysis and multiple correspondence analysis. These assessments resulted in two items being removed from the Practices 2 scale and three items being removed from the knowledge scale.

As soon as an item was removed in any of the steps described above, the process was iterated until all items in each scale fulfilled the criteria. For the final scales, 98.4, 90.0 and 97.0% of the variance could be explained by the first dimension for the Practices 1, Practices 2 and Knowledge scales, respectively. Together with the ratios between first and second Eigenvalue being 15.1, 11.1 and 11.3, these variance percentages were considered sufficient to assume unidimensionality of the scales (32).

The number of items and the CA for the final scales were: Practices 1—eight and CA 0.8079; Practices 2—nine and CA 0.7200; and Knowledge—seven and CA 0.8206. The final set of items included in each scale are listed in Supplementary Table S9.

**Figure 2:**
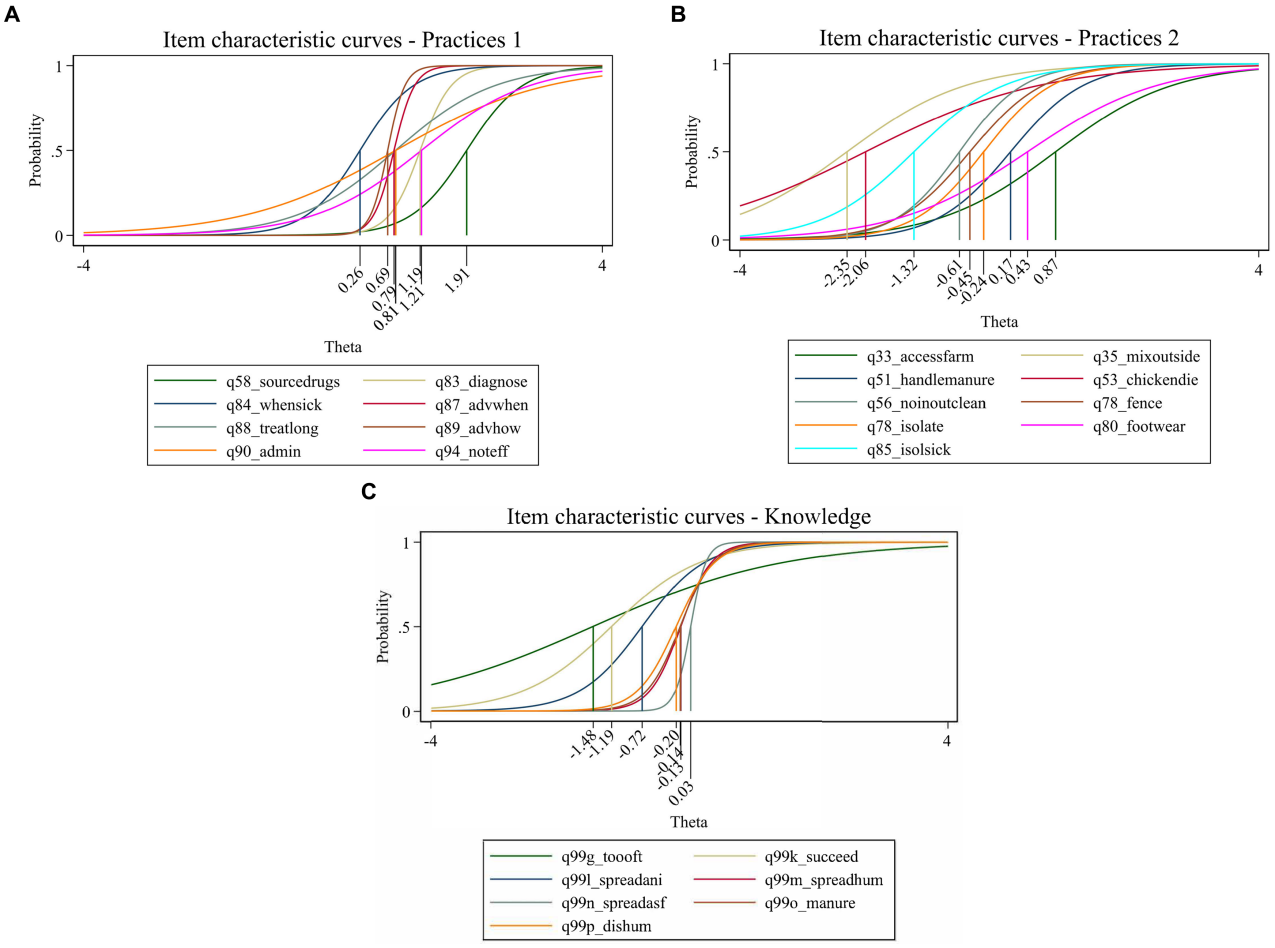
**(A)** Item characteristic curves (ICCs) for Practices 1 scale based on responses in a questionnaire distributed among small- and medium-scale chicken farmers in Thai Nguyen City, Dong Hy and Vo Nhai districts in Vietnam. Practices 1 = Ability to perform desirable practices related to disease management and treatment with antibiotics. Ability and item difficulty is measured in theta (X-axis). See Supplementary Table S9 for full questions. **(B)** Item characteristic curves (ICCs) for Practices 2 scale based on responses in a questionnaire distributed among small- and medium-scale chicken farmers in Thai Nguyen City, Dong Hy and Vo Nhai districts in Vietnam. Practices 2 = Ability to perform desirable practices regarding disease prevention. Ability and item difficulty is measured in theta (X-axis). See Supplementary Table S9 for full questions. **(C)** Item characteristic curves (ICCs) for Knowledge scale based on responses in a questionnaire distributed among small- and medium-scale chicken farmers in Thai Nguyen City, Dong Hy and Vo Nhai districts in Vietnam. Knowledge = Knowledge about antimicrobial resistance (AMR) development and spread. Ability and item difficulty is measured in theta (X-axis). See Supplementary Table S9 for full questions.

Three 2pl models were fitted including the subsets of items generated above. Range in discrimination for the items in each scale were: Practices 1, 0.86–7.55; Practices 2, 0.74–2.02; and Knowledge, 0.68–8.47. The difficulty of the items ranged between: Practices 1, 0.26–1.9; Practices 2, −2.35 to 0.87; and Knowledge, −1.48 to 0.027. Discrimination and difficulty are visualized in the item characteristic curves (ICCs) in Figures 2A–C, where steeper slopes illustrates higher discrimination and difficulty is defined by theta on the X-axis. All discrimination and difficulty values are presented in Supplementary Table S9.

##### 2.5.2.3 Scale evaluation

Test information functions (TIFs) were generated (see Supplementary Figures S1A–C) to evaluate where the three scales were the most informative, i.e., how much information the test gives at different thetas. The Practices 1 scale gives the most information around theta 0.7, the Practices 2 scale around theta −0.5 and the Knowledge scale around theta 0.

##### 2.5.2.4 ANOVA and mixed-effects linear regression

After the fitting of the 2pl models, theta values for practice ability and knowledge were generated for each respondent and each scale. Thereafter, mean theta values for different groups of eight selected variables of interest were calculated. The variables for which the effect on theta was evaluated were: district, respondent’s sex, respondent’s age, respondent’s experience in keeping chickens/hens, respondent’s education level, main reason for keeping chicken/hens, farm size and access to animal health services. The mean theta values between groups were compared through univariable analysis using one-way ANOVA. The effect of the predictor variables on the response variable theta were then further evaluated using multilevel mixed-effects linear regression.

Before fitting the mixed-effects models, causal diagrams were produced including the variables of interest listed in the section above (45). One diagram was produced for the two practice scales and one for the knowledge scale.

The decision on which variables to include in the models was based on the causal diagrams together with evaluation of coefficient change when removing variables, where a change of >25% resulted in the variable being assumed to be a confounder and thus kept in the model. Commune and village were set as random-effects parameters.

Variables affecting the theta value were determined by evaluating *p*-values, where a *p*-value <0.05 was considered significant. All regression models were evaluated with standard visual post estimation methods for residuals, i.e., checks for heteroscedasticity and normality through scatter plots and QQplots.

## 3 Results

### 3.1 Descriptive statistics

#### 3.1.1 Demographics and farm characteristics

Out of the 305 respondents, more than half were male. The mean respondent age was around 50 years with a majority of respondents being between 41 and 60 years old. Almost all respondents were either the household head or spouse of the household head. The mean experience in chicken farming was slightly more than 20 years. Regarding education, the most common was to have completed secondary school. Only five respondents never went to school and six had education from university or college. The main demographics and farm characteristics are presented in Table 3 (for more details, see Supplementary material S2).

**Table 3:** Main demographics and farm characteristics among small- and medium-scale chicken farmers in Thai Nguyen City, Dong Hy and Vo Nhai districts in Vietnam (*n* = 305).

Item	Option	% (number)
Respondent’s sex	Female	39.0 (118)
	Male	61.0 (187)
Education level of respondent	Never went to school	1.6 (5)
	Primary school	25.9 (79)
	Secondary school	43.0 (131)
	High school	27.5 (84)
	College/University	2.0 (6)
	Higher education (e.g., master, PhD)	0 (0)
Main source of income for the household	Crop farming	79.7 (243)
	Self-employment other than farming	6.2 (19)
	Salaried employment off farm	5.3 (16)
	Livestock keeping other than poultry	3.6 (11)
	Poultry keeping (layer and/or broiler)	2.3 (7)
	Poultry keeping (other)	2.0 (6)
	Casual labouring	0.3 (1)
Main reason for keeping chickens/hens	Household consumption	72.1 (220)
	Commercial	23.6 (72)
	Other	4.3 (13)
**Item**		**Number**
Mean age of respondent (years)		51.4
Mean farming experience (years)		21.7
Mean number of chickens/hens (heads)		90.7

The most common main income source for the household was crop farming, while almost no respondents stated that poultry farming (layer and/or broiler) served as the main income source. Further, almost three out of four respondents stated that they kept layers or broilers for household consumption and not for commercial purposes. None of the farms had hired workers. Live animals and animal products (eggs and meat) were most commonly sold to neighbours, friends and family. The mean and median number of chickens/hens kept was 90.7 and 70, respectively.

Farms often kept more than one type of poultry. The most commonly kept species was broiler chickens, followed by dual purpose chickens/hens and layer hens. Regarding other animal species, dogs were the most common, followed by cats, other poultry and pigs. At almost all farms, at least one other species than chickens/hens was kept.

#### 3.1.2 Farm management routines

Chickens/hens of all purposes were most commonly kept fenced outdoors and at a majority of the farms, other animal species at the farm had access to the areas where chickens/hens were kept. However, more than four out of five respondents stated that their chickens/hens never mixed with animals from outside the farm. More than four out of five respondents who said that they slaughtered chickens/hens at the farm stated that they had a specific slaughter area separated from live animals. For more details, see Supplementary material S2.

The most common feed used was grains or crops grown at the farm or locally, and at about two out of five farms, pre-mix or commercial feed was used to some extent. One quarter of respondents added medicines to the feed, and of those, a majority said they added antibiotics.

At three out of four farms, the manure from the chickens/hens was used, sold or given away as fertilizer, and about one third did this without prior treatment or composting. The most common practice when handling diseased chickens/hens was to bury them in the ground.

Using an all-in/all-out system was only practiced in a few farms. At farms where all-in/all-out system was not used, it was most common to remove litter and manure and to clean and disinfect once a month or more seldom.

#### 3.1.3 Disease issues

The most common disease issues among the chickens/hens at the farms one year prior to the study were digestive/intestinal and respiratory diseases. These disease types were also the most common in the cases when respondents had experienced that medicines did not work. Almost no respondents stated that they kept records of disease and mortality among their chickens/hens. For more details, see Supplementary material S2.

#### 3.1.4 Access to pharmaceuticals and animal health services

Almost all respondents said they had the possibility to buy pharmaceuticals or veterinary drugs in their local community (see Table 4), and a large majority of those stated that the most common was to buy them over-the-counter in a veterinary drug shop (see Table 1, details in Supplementary material S2). Of the respondents who sometimes used a veterinary drug shop as a drug source, almost none usually obtained a prescription from a veterinarian before buying medicines. Further, more than four out of five said that the person working in the shop usually does not ask for a prescription before selling.

**Table 4:** Access to pharmaceuticals among small- and medium-scale chicken farmers in Thai Nguyen City, Dong Hy and Vo Nhai districts in Vietnam.

Question	Option	% (number)
Do you have access to buying pharmaceuticals/veterinary drugs in your local community? (*n* = 305)	Yes	96.4 (294)
If you buy veterinary medicines at a veterinary drug shop, do you usually get a prescription from a veterinarian before you buy them? (*n* = 294)	No	93.9 (276)
If you buy veterinary medicines at a veterinary drug shop, does the person working there usually ask for a prescription before selling veterinary medicines to you? (*n* = 294)	No	85.0 (250)

One third of respondents stated that they had access to animal health service providers that give professional advice and help with diagnosis and treatments, and almost no farms belonged to a farmers’ association (see Table 5). Further, almost none was a part of any animal health program that provided routine monitoring and advice, or vaccinations. Of the respondents that said they had access to animal health services, the most commonly accessed service was a veterinary drug shop worker who was not a veterinarian, followed by governmental and private veterinarians (see Table 1, details in Supplementary material S2). A majority of the farmers that had access to animal health services said that they mostly, or sometimes, used the services for advice and/or treatment. Out of those, it was most common to use the services of a veterinary drug shop worker who was not a veterinarian. Of the farmers with access to animal health services, one third stated that the service included laboratory testing and/or autopsies.

**Table 5:** Access to animal health services among small- and medium-scale chicken farmers in Thai Nguyen City, Dong Hy and Vo Nhai districts in Vietnam.

Question	Option	% (number)
Do you have access to animal health service providers that give professional advice on how to handle and prevent diseases among your chickens/hens and help with diagnosis and treatments? (*n* = 305)	Yes	31.8 (97)
If you have access to animal health service providers, which one(s)? (multiple choice) (*n* = 97)	Veterinary drug shop worker (not veterinarian)	62.9 (61)
	Governmental veterinarian	32.0 (31)
	Private veterinarian	15.5 (15)
	Staff of drug company	2.1 (2)
If you have access to animal health services, does the service include laboratory testing and/or autopsies when your chickens/hens are sick? (*n* = 97)	Yes	32.0 (31)
	No	41.2 (40)
	I do not know	26.8 (26)
Is your farm a part of any farmers’ association? (*n* = 305)	No	94.8 (289)
Is your farm a part of any animal health program where you get routine monitoring and advice on the health of your chickens/hens? (*n* = 305)	No	99.0 (302)
Is your farm a part of any animal health program that provides vaccinations for your chickens/hens? (*n* = 305)	No	97.7 (298)

#### 3.1.5 Disease prevention

A majority of respondents said that they, to prevent their chickens/hens from becoming sick, fenced them, isolated/quarantined newly bought animals, and vaccinated against one or several diseases (see Table 1, details in Supplementary material S2). The most common diseases to vaccinate against were Newcastle disease, pasteurellosis (fowl cholera) and Gumboro disease (see Supplementary material S2 for details). A majority of respondents stated that they gave antibiotics as a disease preventive measure.

Handwashing after visiting the animal areas was performed by almost all respondents, while handwashing before entering animal areas was less common. Two out of five farmers had separate footwear that were only used in the areas where the chickens/hens were kept.

#### 3.1.6 Disease management and antibiotic routines

The most common practice when chickens/hens became sick was to give them medicine(s) from a veterinary drug shop, and second most common was to consult a private veterinarian (see Table 1, details in Supplementary material S2). Four out of five farmers said that they usually isolated chickens/hens that became sick from the rest of the poultry in the flock.

For diagnosing disease among the chickens/hens, it was most common for the farmer to do it him−/herself, while one third had a veterinary drug shop worker, who was not a veterinarian, do it. Consulting a private or governmental veterinarian was far less common.

The most commonly used channel for advice regarding both when, and how, to use antibiotics was a veterinary drug shop worker who was not a veterinarian (see Table 1, details in Supplementary material S2). One out of four farmers stated that they used their own judgment for when to use antibiotics, and one out of five that they used their own judgment how to do it. Antibiotic treatment of sick chickens/hens was most commonly provided to sick chickens/hens until they were cured, while about one quarter stated that the treatment length was based on the instructions of a private veterinarian.

It was most common that the farmer him-/herself administered the antibiotics to the animals, either by own experience, or after instructions from a private veterinarian. A majority of respondents said that they never gave a higher or lower dose of antibiotics, or stopped giving antibiotics earlier, than recommended. About one third of respondents said that they often or sometimes gave human medicines to their chickens/hens when they became sick, while giving antibiotics to make the animals grow faster/better or to lay more eggs was almost non-existent practices. Further, almost no farmers kept records of the use of medicines for the chickens/hens.

The most common practices when antibiotic treatment was not effective was to switch to another type of medicine or to go back to the veterinary drug shop for advice from a veterinary drug shop worker, who was not a veterinarian (see Table 1, details in Supplementary material S2). A majority of respondents would handle expired or leftover antibiotics by throwing them into the trash or latrine.

#### 3.1.7 Knowledge about antibiotics and AMR

The results regarding knowledge about antibiotics and AMR are presented in Table 2, with options grouped into correct or incorrect from an AMR development and spread perspective (for details, see Supplementary material S2).

A majority of respondents believed that antibiotics are supposed to be used for treating sick animals, while one third believed that antibiotics are supposed to be used for preventing disease or making animals grow faster/better, solely or in combination with treatment of sick animals. Further, a majority of respondents stated that they thought antibiotics can treat diseases caused by bacteria, and that different antibiotics are needed for different diseases. However, almost half of the respondents did not know that antibiotics cannot be used to treat viral diseases or all kinds of diseases. Further, three out of five did not know that antibiotics are not the same as anti-inflammatory drugs.

A majority of respondents were aware that using antibiotics too often could make diseases difficult to treat in the future and that bacteria could become resistant to antibiotics. However, a majority did not know that the treated animals themselves, and viruses, do not become resistant to antibiotics.

More than half of the respondents knew that resistant bacteria can spread from one animal to another, between humans and animals, and through manure from animals. However, the proportion of respondents that believed resistant bacteria could spread through animal-source foods (ASFs) was lower. While a majority of respondents answered that using too much antibiotics in animals can make it more difficult to treat some diseases in humans, three out of four did not know that antibiotic resistance in human bacteria is not only linked to antibiotic use in humans.

### 3.2 Item response theory

#### 3.2.1 Univariable analysis

The results from the one-way ANOVA conducted after the generation of the three IRT scales (Practices 1, relating to disease management and antibiotic treatment; Practices 2, relating to disease prevention; and Knowledge) are found in Supplementary Table S10.

#### 3.2.2 Multilevel mixed-effects linear regression

The results from the multilevel mixed-effects linear regression are found in Table 6.

**Table 6:** Multi-level mixed effects regression for Practices 1, Practices 2 and Knowledge scales based on a questionnaire distributed among small- and medium-scale chicken farmers in Thai Nguyen City, Dong Hy and Vo Nhai districts in Vietnam.

Variable	Level	Theta mean Practices 1			Theta mean Practices 2			Theta mean Knowledge		
		Coefficient	*p*-value	95% CI	Coefficient	*p*-value	95% CI	Coefficient	*p*-value	95% CI
Constant		1.084	0.000	0.506–1.661	−0.765	0.032	−1.465 to −0.065	−0.609	0.072	−1.272 to 0.054
Fixed effects										
District	Thai Nguyen City	Ref.			Ref.			Ref.		
	Dong Hy	−0.676	0.000*	−1.042 to −0.310	−0.536	0.047*	−1.066 to −0.007	−0.350	0.152	−0.829 to 0.129
	Vo Nhai	−0.789	0.000*	−1.099 to −0.478	−0.212	0.381	−0.687 to 0.263	−0.215	0.317	−0.638 to 0.207
Respondent’s sex	Female	−0.132	0.141	−0.308 to 0.044	0.251	0.007*	0.069–0.432	0.127	0.162	−0.051 to 0.304
	Male	Ref.			Ref.			Ref.		
Age of respondent	21–30	0.484	0.064	−0.028 to 0.996	−0.533	0.069	−1.109 to 0.043	−0.230	0.381	−0.744 to 0.284
	31–40	0.077	0.580	−0.195 to 0.348	−0.380	0.036*	−0.734 to −0.025	−0.045	0.747	−0.315 to 0.226
	41–50	Ref.			−0.073	0.651	−0.388 to 0.242	Ref.		
	51–60	0.028	0.815	−0.205 to 0.260	−0.306	0.026*	−0.576 to −0.036	−0.094	0.432	−0.327 to 0.140
	>60	0.041	0.792	−0.262 to 0.344	Ref.			−0.101	0.522	−0.411 to 0.209
Livestock keeping experience of respondent (years)	1–10	Ref.			Ref.			Ref.		
	11–20	−0.309	0.020*	−0.568 to −0.049	−0.067	0.627	−0.337 to 0.203	−0.086	0.525	−0.350 to 0.178
	21–30	−0.260	0.096	−0.565 to 0.046	−0.013	0.937	−0.331 to 0.306	−0.059	0.714	−0.372 to 0.255
	31–40	−0.430	0.023*	−0.801 to −0.059	−0.075	0.703	−0.461 to 0.311	−0.044	0.822	−0.423 to 0.336
	>40	−0.383	0.205	−0.975 to 0.209	−0.226	0.467	−0.834 to 0.383	0.321	0.292	−0.276 to 0.917
Education level of respondent	Never went to school	−0.324	0.337	−0.985 to 0.338	−0.556	0.098	−1.215 to 0.103	−0.353	0.283	−0.999 to 0.292
	Primary school	−0.088	0.479	−0.332 to 0.156	Ref.			Ref.		
	Secondary school	−0.070	0.492	−0.271 to 0.130	0.287	0.015*	0.057–0.517	0.374	0.001*	0.148–0.601
	High school	Ref.			0.467	0.000*	0.212–0.722	0.557	0.000*	0.306–0.808
	College/University	−0.151	0.631	−0.767 to 0.465	0.752	0.017*	0.133–1.371	1.118	0.000*	0.511–1.726
Main reason for keeping chickens/hens	Commercial	Ref.			Ref.			Ref.		
	Household consumption	0.168	0.142	−0.056 to 0.392	0.322	0.006*	0.091–0.553	0.281	0.015*	0.055–0.508
	Other	0.400	0.084	−0.054 to 0.854	0.033	0.889	−0.434 to 0.501	−0.479	0.041*	−0.937 to −0.021
Farm size	<100	Ref.			Ref.			Ref.		
	≥100	0.049	0.645	−0.158 to 0.255	0.453	0.000*	0.228–0.678	0.306	0.007*	0.083–0.529
Access to animal health services	Yes	0.443	0.000*	0.245–0.641	−0.166	0.119	−0.375 to 0.043	−0.250	0.018*	−0.457 to −0.043
	No	Ref.			Ref.			Ref.		
Random effects		Estimate			Estimate			Estimate		
Commune		<0.001		-	0.040		0.005–0.311	0.019		0.001–0.465
Village		0.063		0.027–0.143	0.123		0.048–0.315	0.149		0.064–0.347

Practices 1 = Ability to perform desirable practices related to disease management and treatment with antibiotics, Practices 2 = Ability to perform desirable practices regarding disease prevention, Knowledge = Knowledge about antimicrobial resistance (AMR) development and spread. *p < 0.05.

For the Practices 1 scale, variables significantly associated with a higher ability to perform desirable practices regarding disease management and treatment with antibiotics were: living in Thai Nguyen City, having short livestock keeping experience, and having access to animal health services.

For the Practices 2 scale, variables significantly associated with a higher ability to perform desirable practices related to disease prevention were: living in Thai Nguyen City, being female, being >60 years, having high education, keeping chickens/hens for household consumption, and having ≥100 chickens/hens.

For the Knowledge scale, variables significantly associated with a higher knowledge about effects and spread of AMR were: having a high education, keeping chickens/hens for household consumption, having ≥100 chickens/hens, and not having access to animal health services.

## 4 Discussion

This study is aimed to increase our understanding of farm level variables associated with small- and medium-scale poultry farmers’ AMR-related practices and knowledge, in an emerging Southeast Asian economy. Similar knowledge- and practice-studies have been performed previously in the region. However, applying IRT as an analytic tool allowed us to investigate each individual’s unobservable measurement of the underlying trait, the probability of different responses to the items in the test, while standardizing the scores of respondents on a standardized scale based on the individual difficulty and quality of the questions (or items) in the test.

The overall picture of the studied farms is that they were of non-commercial character, not specialized in chicken production, but with highly educated and experienced farmers. Even though the chicken farming was not mainly for commercial purposes for most of the farmers during the time of the study, selling activities were still common, e.g., almost two thirds of the farmers sold live chickens, although mainly to people closest to them (neighbours/friends/family). However, according to personal communications, the proportion of farms keeping chickens for household consumption might have been overestimated since the study was conducted during the Covid-19 pandemic when commercial poultry raising became more difficult. Hence, it is possible that some farms previously had a more commercial character.

Almost all farmers had the possibility to buy pharmaceuticals or veterinary drugs in their local community and, as in many LMICs (18), a prescription was almost never required when buying them. On the other hand, only one third of the respondents stated that they had access to professional animal health services. In addition, other supportive systems like farmers’ associations or animal health programs were reportedly either not available or not used. The combination of high access to drugs and low access to animal health services raises concerns about the risk for inappropriate use of antibiotics and subsequent AMR development. These concerns are further strengthened by the fact that almost two thirds of respondents stated that they give antibiotics to prevent their chickens from becoming sick, which is in line with several previous studies in Vietnamese poultry farms (12, 13, 23).

To prevent disease, a majority of farmers implemented both biosecurity measures, such as isolation of newly bought and sick animals, and vaccination strategies. However, the most common disease preventive measure was administration of antibiotics which, as mentioned above, is problematic from an AMR development perspective.

To use antibiotics as growth promoters is a common practice in livestock production, including poultry farming, in many parts of the world (3, 46, 47). However, in the surveyed farms this practice seemed non-existent, which is beneficial from an AMR-mitigation perspective. Similar results have also been presented in another study on Vietnamese poultry farms (13). It is possible that this absence of use of antibiotics as growth promoters is related to the ban of such use according to Vietnamese legislation (17). The common practice of throwing expired or leftover antibiotics into the trash or latrine, is however, concerning, as this imposes a risk for antibiotic contamination of the environment (48).

In cases of disease among the chickens in the visited farms, the most common first response was to give medicines from a veterinary drug shop, in contrast to results from a previous study on chicken farms in northern Vietnam where it was most common to seek advice from a veterinarian (13). However, in that study the farms were larger and more commercially oriented, which might explain this difference.

Diagnosis of sick chickens was most commonly made by the farmers themselves, or less commonly by a veterinary drug shop worker. Similarly, advice on when and how to use antibiotics was most commonly obtained from a veterinary drug shop worker, or from the farmer’s own judgment. A high dependence on veterinary drug shop workers for advice regarding antimicrobial use has been shown in previous studies on Vietnamese poultry farms (12, 23, 49), as well as for other farmed species in Asian countries (50–53). Depending on the knowledge and experience of the farmer and drug shop worker, these practices can have implications on the appropriateness and success of each case of antibiotic treatment.

Also, the non-existent use of records for disease, mortality and drug use might further complicate the farmers’ ability to handle future disease events appropriately. Record keeping has previously been shown to be associated with higher knowledge about AMR and more favourable AMR-related practices among poultry farmers in Vietnam (13), and in several African countries (15).

In the multi-variable analysis, there were only three variables that showed significant association with the ability to perform desirable practices related to disease management and treatment with antibiotics (Practices 1). As expected, access to animal health services was one of them, since the questions included in this scale to a large extent are connected to the use of animal health services for disease handling and treatment. Geographic district was also significantly associated with the level of practice, indicating some regional effects.

Thirdly, livestock-keeping experience was significantly associated with the Practices 1 score. At first glance, it is surprising that respondents with a livestock-keeping experience of one to ten years had higher scores than respondents with an experience of 11–20, and 31–40 years, respectively. However, a person who is new to farming might have a more up-to-date education on how to handle disease. Being inexperienced, they might also be more prone to seek professional advice. Contrary to findings from a previous study among Vietnamese poultry farmers (23), and several studies from other countries (52, 54), education level was not significantly associated with the level of disease management and antibiotic treatment practices. However, comparisons of studies are generally difficult because of the use of different questionnaires, different variables used for analysis, and variations in statistical methods.

For the second practice scale, evaluating disease prevention ability (Practices 2), the picture is more complex with significant associations for six variables. Of the three scales, this was the only one where gender and age were significantly associated with the score, but the reasons why these variables would affect the level of disease prevention ability are unclear. Previously, an association between ABU-related practices and gender has been shown in Vietnam (13). More expected was the relationship between education and practice level, where people with higher education performed good disease prevention practices to a higher degree.

That having a large farm (>100 birds) was associated with a higher disease prevention score can be explained by the fact that larger numbers of birds often is associated with higher animal density and consequent higher disease pressure, making disease preventive measures important. Owners of large farms are also more likely to sell birds for income, which makes disease prevention important from an economic perspective. However, with that reasoning, it was unexpected that keeping chickens for household consumption was associated with higher scores for this scale. As stated above, it is suspected that some farms in the household consumption group actually kept chickens for commercial purposes before the Covid-19 pandemic. Hence, this relationship should be interpreted with caution.

The results for the two practice scales show that interventions might need tailoring to different target groups depending on which practices that need to be improved. If the focus is on disease management and antibiotic treatment, the target group would appear to be experienced farmers in the more rural areas of Thai Nguyen province, and who have low access to animal health services. On the other hand, if focus is on improving disease preventive practices, young to middle-aged, male, small-scale farmers with low education level should be prioritized. This illustrates the great difficulty in targeting interventions, having to consider multiple variables that might affect different areas of the AMR-related field in different ways.

Four variables were significantly associated with the respondents’ knowledge scores (Knowledge scale). Most surprising was the finding that not having access to animal health services was associated with a higher score, contrary to the finding for the practice scale related to disease management and antibiotic treatment practices (Practices 1). One could reason that without access to animal health services, you need to rely on your own knowledge to a larger extent, and therefore have an incitement to learn more about disease handling and treatment, including ABU. Another theory might be that animal health professionals would be hesitant to share their knowledge because they want farmers to rely on their services. However, these are only speculations that need to be investigated further.

That two of the other variables, high education level and large farm size, were significantly associated with higher knowledge scores was more expected. For example, an association between knowledge and education level has previously been shown within poultry production in different LMICs, including in Vietnam (13, 52, 54). Further, it is reasonable that farmers with more animals are closer to commercialization or intensification of their farming methods, making it more important for them to learn about handling disease. If keeping chickens for household consumption is actually a variable to consider here is difficult to evaluate, as for the Practices 2 scale discussed above.

The descriptive results for the knowledge questions reveal that the knowledge level overall is quite high in some areas, but that there are also knowledge gaps to be addressed. On the positive side, more than two thirds of respondents stated that antibiotics are supposed to be used for disease treatment only, and almost 90% understood that antibiotics can treat bacterial disease. On the other hand, almost half of respondents also said that antibiotics can be used to treat all kinds of diseases, including viral diseases. Several questions revealed that a majority knew about AMR, even though an understanding of how AMR spreads was often lacking. These results could possibly be used as guidance if educational interventions are considered in the future. A suggestion would then be to focus on how resistance can spread, both between animals and between animals and humans. The connections between AMR in animals and humans should also be emphasized. Furthermore, results from the regression analysis suggest that there should be a focus on small-scale farmers, and farmers with a low education level in the Thai Nguyen province.

When comparing the three investigated scales, similarities were mainly found between the disease prevention practices scale (Practices 2) and the knowledge scale. It might have been expected to find more similarities between the practice scale related to disease management and antibiotic treatment (Practices 1), and the knowledge scale. Several previous studies among poultry farmers in LMICs have shown a positive correlation between knowledge about antibiotics and AMR and good ABU practices (13, 15, 52, 54). However, it has also been shown that good knowledge does not necessarily translate into desirable practices (15). It is also noteworthy that long experience of keeping livestock showed no significant positive association with the scores for any of the three scales, as has been shown in some other studies (15, 52).

To the authors’ knowledge this is the first study to use IRT as a method to evaluate farmers’ knowledge and practices related to ABU and AMR. IRT has several advantages over simpler statistical methods, primarily generating more reliable results. In addition, with IRT each question is automatically evaluated regarding its quality and difficulty, providing valuable information for adjusting the questionnaire for future use. However, IRT demands more preparatory work, as well as more time for data processing and statistical analyses, which should be considered in the planning stage. As more studies using IRT are performed within the AMR field, this time can be reduced, hopefully, as sets of questions that are proved to work well, such as the three scales in this study, are generated. Today, IRT is still uncommon within veterinary public health research, so comparisons of results with other studies’ are difficult.

The current study has revealed several parameters to consider when planning interventions to reduce AMR in livestock production in LMICs. The wide range of characteristics that may impact farmers’ knowledge and practices calls for further work in Vietnam, and other Southeast Asian countries. Further, studies to decide suitable and effective interventions are also required.

The results from this study show the important role of veterinary drug shop workers in the farmers’ local communities. Their knowledge and routines regarding ABU and AMR are likely to affect ABU and AMR development at the farm level. Therefore, it is of great interest to investigate the knowledge and practices in the AMR area among these veterinary drug shop workers. Combining the results generated from this study, and similar ones, with a study among veterinary drug shop workers would generate a better understanding for the context in which small- and medium-scale farmers operate.

## 5 Conclusion

By applying the IRT approach, this study demonstrates the complexity in understanding what drives farmers’ behaviour in relation to ABU and AMR in an area with high access to veterinary drugs, low access to veterinary health services and high use of antibiotics for disease prevention. Overall, there were more similarities in significantly associated variables between the disease prevention practices scale and the knowledge scale, than between the disease management and antibiotic treatment practices scale and the knowledge scale. Yet, differences were seen between all three scales. These thorough analyses of variables impacting farmers’ practices and knowledge illustrate that perfect targeting of interventions is challenging. When planning for interventions you need to understand the context to decide which variables to focus on in each specific setting, both in terms of access to veterinary drugs and veterinary health services, but also other farm characteristics. This in turn may be one of the reasons why change to better AMR-related practices takes time, there is no one-size-fits-all solution.

### 5.1 Limitations of the study

In this study, official registers of poultry farms were used as sampling frames. When the field work was initiated, it was noticed that there were over-coverage in the frames since many households did not keep chicken anymore, or kept a different number than registered. This negatively impacted the randomness of the sample since replacements households were not selected randomly. For all districts, there was a larger proportion of small-scale farms that needed to be replaced than medium-scale farms, making the randomness in the sample of small-scale farms lower in comparison. Also, the over-coverage probably affected the stratification since the proportion of replaced households differed between districts. Under-coverage of the frames is also possible if not all small-scale farmers are in the official registers.

Another factor to consider is the risk for different kinds of biases when performing questionnaire-based surveys, e.g., desirability and recall bias. Further, 43 of the prospective respondents did not agree to participate, which means that there might be some nonresponse bias.

## Data availability statement

The original contributions presented in the study are included in the article/Supplementary material, further inquiries can be directed to the corresponding author. The original contributions presented in the study are publicly available. This data can be found here: https://hdl.handle.net/20.500.12703/4023.

## Ethics statement

The studies involving humans were approved by Institutional Review Board of Hanoi School of Public Health (HUPH). The studies were conducted in accordance with the local legislation and institutional requirements. The participants provided their written informed consent to participate in this study.

## Author contributions

SN: Conceptualization, Data curation, Formal analysis, Investigation, Methodology, Project administration, Visualization, Writing – original draft, Writing – review & editing. TN-T: Investigation, Methodology, Project administration, Resources, Supervision, Writing – review & editing. HX: Investigation, Project administration, Resources, Supervision, Writing – review & editing. JL: Formal analysis, Methodology, Validation, Writing – review & editing. SB: Conceptualization, Methodology, Validation, Writing – review & editing. JJ: Conceptualization, Methodology, Validation, Writing – review & editing. UM: Conceptualization, Funding acquisition, Methodology, Project administration, Resources, Supervision, Validation, Writing – review & editing.

## References

[1] Holmes AH Moore LSP Sundsfjord A Steinbakk M Regmi S Karkey A. Understanding the mechanisms and drivers of antimicrobial resistance. Lancet. (2016) 387:176–87. doi: 10.1016/S0140-6736(15)00473-0 26603922

[2] FAO. Drivers, dynamics and epidemiology of antimicrobial resistance in animal production. Rome: Food and Agriculture Organization of the United Nations (2016) ISBN 978-92-5-109441-9.

[3] Marshall BM Levy SB. Food animals and antimicrobials: impacts on human health. Clin Microbiol Rev. (2011) 24:718–33. doi: 10.1128/cmr.00002-11, PMID: 21976606 PMC3194830

[4] Bengtsson B Greko C. Antibiotic resistance--consequences for animal health, welfare, and food production. Ups J Med Sci. (2014) 119:96–102. doi: 10.3109/03009734.2014.901445, PMID: 24678738 PMC4034566

[5] World Bank. Drug-resistant infections: A threat to our economic future. Washington, DC: World Bank (2017) License: Creative Commons Attribution CC BY 3.0 IGO.

[6] Van Boeckel TP Brower C Gilbert M Grenfell Bryan T Levin Simon A Robinson Timothy P. Global trends in antimicrobial use in food animals. Proc Natl Acad Sci. (2015) 112:5649–54. doi: 10.1073/pnas.1503141112, PMID: 25792457 PMC4426470

[7] Van Boeckel TP Pires J Silvester R Zhao C Song J Criscuolo NG. Global trends in antimicrobial resistance in animals in low- and middle-income countries. Science. (2019) 365. doi: 10.1126/science.aaw1944, PMID: 31604207

[8] Aarestrup FM Wegener HC Collignon P. Resistance in Bacteria of the food chain: epidemiology and control strategies. Expert Rev Anti-Infect Ther. (2008) 6:733–50. doi: 10.1586/14787210.6.5.733 18847409

[9] da Costa PM Loureiro L Matos AJ. Transfer of multidrug-resistant Bacteria between intermingled ecological niches: the Interface between humans, animals and the environment. Int J Environ Res Public Health. (2013) 10:278–94. doi: 10.3390/ijerph10010278, PMID: 23343983 PMC3564142

[10] ECDC (European Centre for Disease Prevention and Control), EFSA (European Food Safety Authority), EMA (European Medicines Agency). ECDC/EFSA/EMA first joint report on the integrated analysis of the consumption of antimicrobial agents and occurrence of antimicrobial resistance in Bacteria from humans and food-producing animals. Stockholm/Parma/London: ECDC/EFSA/EMA; 2015. EFSA J. (2015) 13:4006:114. doi: 10.2903/j.efsa.2015.4006

[11] Gilbert M Conchedda G Van Boeckel TP Cinardi G Linard C Nicolas G. Income disparities and the global distribution of intensively farmed chicken and pigs. PLoS One. (2015) 10:e0133381. doi: 10.1371/journal.pone.0133381, PMID: 26230336 PMC4521704

[12] Carrique-Mas JJ Trung NV Hoa NT Mai HH Thanh TH Campbell JI. Antimicrobial usage in chicken production in the Mekong Delta of Vietnam. Zoonoses Public Health. (2015) 62:70–8. doi: 10.1111/zph.12165, PMID: 25430661

[13] Pham-Duc P Cook MA Cong-Hong H Nguyen-Thuy H Padungtod P Nguyen-Thi H. Knowledge, attitudes and practices of livestock and aquaculture producers regarding antimicrobial use and resistance in Vietnam. PLoS One. (2019) 14:e0223115. doi: 10.1371/journal.pone.0223115, PMID: 31553776 PMC6760827

[14] Zellweger RM Carrique-Mas J Limmathurotsakul D Day NPJ Thwaites GE Baker S. A current perspective on antimicrobial resistance in Southeast Asia. J Antimicrob Chemother. (2017) 72:2963–72. doi: 10.1093/jac/dkx260, PMID: 28961709 PMC5890732

[15] Caudell MA Dorado-Garcia A Eckford S Creese C Byarugaba DK Afakye K. Towards a bottom-up understanding of antimicrobial use and resistance on the farm: a knowledge, attitudes, and practices survey across livestock Systems in Five African Countries. PLoS One. (2020) 15:e0220274. doi: 10.1371/journal.pone.0220274, PMID: 31978098 PMC6980545

[16] Malijan GM Howteerakul N Ali N Siri S Kengganpanich M Nascimento R. A scoping review of antibiotic use practices and drivers of inappropriate antibiotic use in animal farms in who Southeast Asia region. One Health. (2022) 15:100412. doi: 10.1016/j.onehlt.2022.100412, PMID: 36277092 PMC9582544

[17] Vietnamese Animal Husbandry Law (Law No. 32/2018/QH14). Hanoi (2018). Available at: https://www.economica.vn/Content/files/LAW%20%26%20REG/Law%20on%20Animal%20Husbandry%202018.pdf (Accessed September 29, 2023).

[18] European Commission, Directorate-General for Health and Food Safety. Non-EU Countries' National Policies and measures on antimicrobial resistance: Overview report. Luxembourg: Publications Office of the European Union (2018).

[19] Carrique-Mas JJ Choisy M Van Cuong N Thwaites G Baker S. An estimation of Total antimicrobial usage in humans and animals in Vietnam. Antimicrob Resist Infect Control. (2020) 9:16. doi: 10.1186/s13756-019-0671-7, PMID: 31956405 PMC6961235

[20] Nguyen NT Nguyen HM Nguyen CV Nguyen TV Nguyen MT Thai HQ. Use of Colistin and other critical antimicrobials on pig and chicken farms in southern Vietnam and its association with resistance in commensal *Escherichia Coli* bacteria. Appl Environ Microbiol. (2016) 82:3727–35. doi: 10.1128/aem.00337-16, PMID: 27084016 PMC4907207

[21] Cuong NV Phu DH Van NTB Dinh Truong B Kiet BT Hien BV. High-resolution monitoring of antimicrobial consumption in Vietnamese small-scale chicken farms highlights discrepancies between study metrics. Front Vet Sci. (2019) 6:174. doi: 10.3389/fvets.2019.00174, PMID: 31294033 PMC6598194

[22] WHO. Critically important antimicrobials for human medicine 6th revision. Geneva: World Health Organization, License: CC BY-NC-SA 3.0 IGO. ISBN 978–92–4-151552-8 (2019).

[23] Luu QH Nguyen TLA Pham TN Vo NG Padungtod P. Antimicrobial use in household, semi-industrialized, and industrialized pig and poultry farms in Viet Nam. Prev Vet Med. (2021) 189:105292. doi: 10.1016/j.prevetmed.2021.105292, PMID: 33621709

[24] Carrique-Mas J Van NTB Cuong NV Truong BD Kiet BT Thanh PTH. Mortality, disease and associated antimicrobial use in commercial small-scale chicken flocks in the Mekong Delta of Vietnam. Prev Vet Med. (2019) 165:15–22. doi: 10.1016/j.prevetmed.2019.02.005, PMID: 30851923 PMC6418316

[25] Nohrborg S Dione MM Winfred AC Okello L Wieland B Magnusson U. Geographic and socioeconomic influence on knowledge and practices related to antimicrobial resistance among smallholder pig farmers in Uganda. Antibiotics. (2022) 11. doi: 10.3390/antibiotics11020251, PMID: 35203853 PMC8868422

[26] Robinson TP Bu DP Carrique-Mas J Fèvre EM Gilbert M Grace D. Antibiotic resistance: mitigation opportunities in livestock sector development. Animal. (2017) 11:1–3. doi: 10.1017/s1751731116001828 27549404

[27] Sadiq MB Syed-Hussain SS Ramanoon SZ Saharee AA Ahmad NI Mohd Zin N. Knowledge, attitude and perception regarding antimicrobial resistance and usage among ruminant farmers in Selangor, Malaysia. Prev Vet Med. (2018) 156:76–83. doi: 10.1016/j.prevetmed.2018.04.013, PMID: 29891148

[28] Ström GH Björklund H Barnes AC Da CT Nhi NHY Lan TT. Antibiotic use by small-scale farmers for freshwater aquaculture in the upper Mekong Delta, Vietnam. J Aquat Anim Health. (2019) 31:290–8. doi: 10.1002/aah.10084, PMID: 31407408

[29] Gemeda BA Amenu K Magnusson U Dohoo I Hallenberg GS Alemayehu G. Antimicrobial use in extensive smallholder livestock farming Systems in Ethiopia: knowledge, attitudes, and practices of livestock keepers. Front Vet Sci. (2020) 7:55. doi: 10.3389/fvets.2020.00055, PMID: 32175334 PMC7055293

[30] Thi Huong-Anh N Van Chinh D Thi T-HT. Antibiotic residues in chickens and farmers' knowledge of their use in Tay Ninh Province, Vietnam, in 2017. Asia Pac J Public Health. (2020) 32:126–32. doi: 10.1177/1010539520909942, PMID: 32174126

[31] Silva GS Leotti VB Castro SMJ Medeiros AAR Silva A Linhares DCL. Assessment of biosecurity practices and development of a scoring system in swine farms using item response theory. Prev Vet Med. (2019) 167:128–36. doi: 10.1016/j.prevetmed.2019.03.020, PMID: 31027714

[32] Dohoo I Emanuelson U. The use of item response theory models to evaluate scales designed to measure knowledge of, and attitudes toward, antibiotic use and resistance in Swedish dairy producers. Prev Vet Med. (2021) 195:105465. doi: 10.1016/j.prevetmed.2021.105465 34419777

[33] Miraballes C Riet-Correa F de Freitas DG Pasternak LG Dohoo I. Design and analysis of a structured questionnaire to assess the probability of elimination of *Rhipicephalus Microplus* from farms in a subtropical area. Prev Vet Med. (2022) 200:105576. doi: 10.1016/j.prevetmed.2022.105576 35038639

[34] General Statistics Office. Results of the 2020 mid-term rural and agricultural survey. Hanoi: Statistical Publishing House (2021).

[35] General Statistics Office. Statistical yearbook of Vietnam 2022. Hanoi: Statistical Publishing House (2023).

[36] General Statistics Office. Results of the rural, agricultural and fishery census 2016. Hanoi: Statistical Publishing House (2018).

[37] Thai Nguyen Statistics Office. Thai Nguyen statistical yearbook 2022. Hanoi: Statistical Publishing House (2023).

[38] General Statistics Office. Completed results of the 2019 Viet Nam population and housing census. Hanoi: Statistical Publishing House (2020).

[39] Research Randomizer. Available at: https://www.randomizer.org/ (Accessed November 29, 2021).

[40] Netigate. Available at: https://www.netigate.net/ (Accessed January 20, 2022).

[41] STATA Statistical Software. STATACorp Ltd, Texas College Station, Texas, version 17.0. Available at: https://www.stata.com/

[42] Hobart J Cano S. Improving the evaluation of therapeutic interventions in multiple sclerosis: The role of new psychometric methods. Health Technol Assess. (2009) 13:1–177. doi: 10.3310/hta13120 19216837

[43] Cronbach LJ. Coefficient alpha and the internal structure of tests. Psychometrika. (1951) 16:297–334. doi: 10.1007/BF02310555

[44] Briggs SR Cheek JM. The role of factor analysis in the development and evaluation of personality scales. J Pers. (1986) 54:106–48. doi: 10.1111/j.1467-6494.1986.tb00391.x

[45] DAGitty. Available at: https://dagitty.net/ (Accessed November 22, 2022).

[46] Laxminarayan R Boeckel T Teillant A. The economic costs of withdrawing antimicrobial growth promoters from the livestock sector. Paris: OECD Publishing (2015) OECD Food, Agriculture and Fisheries Papers, No. 78.

[47] Coyne L Arief R Benigno C Giang VN Huong LQ Jeamsripong S. Characterizing antimicrobial use in the livestock sector in three south east Asian countries (Indonesia, Thailand, and Vietnam). Antibiotics (Basel). (2019) 8. doi: 10.3390/antibiotics8010033, PMID: 30934638 PMC6466601

[48] Samreen AI Malak HA Abulreesh HH. Environmental antimicrobial resistance and its drivers: a potential threat to public health. J Glob Antimicrob Resist. (2021) 27:101–11. doi: 10.1016/j.jgar.2021.08.001, PMID: 34454098

[49] Truong DB Doan HP Doan Tran VK Nguyen VC Bach TK Rueanghiran C. Assessment of drivers of antimicrobial usage in poultry farms in the Mekong Delta of Vietnam: a combined participatory epidemiology and Q-sorting approach. Front Vet Sci. (2019) 6:84. doi: 10.3389/fvets.2019.00084, PMID: 30968033 PMC6442645

[50] Hallenberg GS Jiwakanon J Angkititrakul S Kang-Air S Osbjer K Lunha K. Antibiotic use in pig farms at different levels of intensification-farmers' practices in northeastern Thailand. PLoS One. (2020) 15:e0243099. doi: 10.1371/journal.pone.0243099, PMID: 33306684 PMC7732346

[51] Heyman J. Antimicrobial drugstore supply for Cambodian livestock farmers - a survey study on retailers’ influence and knowledge of antimicrobial resistance [Master’s thesis on the internet]. Uppsala: Swedish University of Agricultural Sciences; (2020). Available at: https://stud.epsilon.slu.se/15855/1/heyman_J_200217.pdf

[52] Hassan MM Kalam MA Alim MA Shano S Nayem MRK Badsha MR. Knowledge, attitude, and practices on antimicrobial use and antimicrobial resistance among commercial poultry farmers in Bangladesh. Antibiotics. (2021) 10. doi: 10.3390/antibiotics10070784, PMID: 34203195 PMC8300693

[53] Huber L Hallenberg GS Lunha K Leangapichart T Jiwakanon J Hickman RA. Geographic drivers of antimicrobial use and resistance in pigs in Khon Kaen Province, Thailand. Front Vet Sci. (2021) 8:659051. doi: 10.3389/fvets.2021.659051, PMID: 33996982 PMC8113701

[54] Moffo F Mouliom Mouiche MM Kochivi FL Dongmo JB Djomgang HK Tombe P. Knowledge, attitudes, practices and risk perception of rural poultry farmers in Cameroon to antimicrobial use and resistance. Prev Vet Med. (2020) 182:105087. doi: 10.1016/j.prevetmed.2020.105087, PMID: 32726706

