# Supplementary material for: Understanding Vietnamese chicken farmers’ knowledge and practices related to antimicrobial resistance using an item response theory approach
Source: Front Vet Sci. 2024 Apr 5;11:1319933. doi: 10.3389/fvets.2024.1319933 (PMC11027563; doi:10.3389/fvets.2024.1319933)
Supplement: Supplementary file 1 [file Data_Sheet_1.PDF]

<u>Supplementary material S1, Questionnaire</u>

Consent to participate:

Information to the respondent: Thank you for participating in this study conducted by the Swedish University of Agricultural Sciences, Sweden, and International Livestock Research Institute (ILRI), Vietnam, and National Institute of Veterinary Research (NIVR), Vietnam. This questionnaire contains questions about your farm characteristics, management routines, access to animal health services and veterinary drugs, disease issues at the farm, prevention and management of disease, routines for treatment with veterinary drugs and finally some questions and statements regarding antibiotics. We would like to emphasize that participating in this study is voluntary and you can retract your consent at any time. The answers collected in this questionnaire will be anonymized and your identity will not be revealed in any published material that results from this questionnaire.

Consent to participate (Yes/No)

General information about the data collection

Information to the enumerator:

The respondent should be the person with the main responsibility for the animal production at the farm.

The questionnaire ID should be the same ID as on the fecal samples (swabs and pooled).

1. Questionnaire ID
2. Date
3. Enumerator's name
    a. AAA AAA
    b. BBB BBB
    c. CCC CCC
    d. DDD DDD
    e. EEE EEE
    f. Other (specify)

4. Enumerator's sex
    a. Female
    b. Male

5. District
    a. Dong Hy
    b. Thai Nguyen
    c. Vo Nhai

6. A) Commune Dong Hy a.-g.

   B) Commune Thai Nguyen a.-d.

   C) Commune Vo Nhai a.-n.

7. Village (free text)

Farm characteristics, general management routines and access to animal health services and veterinary drugs

Information to the respondent: This section includes questions about you and your farm, what animals and type of production you have, and which general management routines you apply. It also deals with the access to professional animal health services and veterinary drugs. These questions are for us to get a better understanding of the conditions and activities at your farm.

Demographics of the farm and respondent

8. Sex of the respondent
   a. Female
   b. Male

9. Age of respondent (in years)

10. What is the main role of the respondent on the farm? (single choice)
    a. Household head
    b. Wife/husband
    c. Child
    d. Parents
    e. Grandparents
    f. Worker
    g. Other relationship (specify)

11. Years of farming experience of the respondent

12. What is the education level of the respondent? (single choice)
    a. Never went to school
    b. Primary school
    c. Secondary school
    d. High school
    e. Vocational training
    f. College/university
    g. Higher education (master, PhD etc.)
    h. Other (specify)

13. What is the **main source** of income for the household? (single choice)
    a. Poultry keeping (broiler chickens and/or layer hens)
    b. Poultry keeping (other)
    c. Livestock keeping other than poultry
    d. Crop farming
    e. Self-employment other than farming
    f. Salaried employment off farm
    g. Casual laboring
    h. I don't know
    i. Other (specify) ….

14. What is the **main reason** for keeping chickens/hens? (single choice)

a. Commercial
b. Household consumption
c. Other (specify) ….

15. How many different people, other than the farmer (manager or owner), work with the chickens/hens?

16. Are there hired workers on the farm?
    a. Yes
    b. No
    c. I don't know

17. A) Do you understand the word antibiotics?
    a. Yes
    b. No

Information to the enumerator:

Provide pictures of the most commonly used antibiotics in the region as examples, both to the respondents who state that they know and that they don't know the word 'antibiotics'.

B) Do you recognize some, or all, of the medicines showed in the pictures?
a. Yes
b. No

Farm setup

18. Do you keep dual purpose chickens/hens (for both meat and eggs)?
    a. No
    b. Yes, how many …

19. Do you keep broiler chickens?
    a. I have only dual purpose chickens/hens
    b. No
    c. Yes, how many …

20. Do you keep layer hens?
    a. I have only dual purpose chickens/hens
    b. No
    c. Yes, how many …

21. Do you keep other poultry species?
    a. No
    b. Yes, how many …

22. Do you keep pigs?
    a. No
    b. Yes, how many …

23. Do you keep cattle?
    a. No

b. Yes, how many …

24. Do you keep small ruminants?
    a. No
    b. Yes, how many …

25. Do you keep horses/donkeys?
    a. No
    b. Yes, how many …

26. Do you keep dogs?
    a. No
    b. Yes, how many …

27. Do you keep cats?
    a. No
    b. Yes, how many …

28. Do you keep rodents/rabbits?
    a. No
    b. Yes, how many …

29. Do you keep any other animal species?
    a. No
    b. Yes, how many and what …

30. How do you keep you dual purpose chickens/hens? (single choice)
    a. Free range outdoors
    b. Fenced outdoors
    c. Free range indoors (housed)
    d. Caged indoors
    e. Free range outdoors during the day, housed at night
    f. Other (specify)

31. How do you keep your broiler chickens? (skip if you ONLY have dual purpose chickens/hens) (single choice)
    a. Free range outdoors
    b. Fenced outdoors
    c. Free range indoors (housed)
    d. Caged indoors
    e. Free range outdoors during the day, housed at night
    f. Other (specify)

32. How do you keep your layer hens? (skip if you ONLY have dual purpose chickens/hens) (single choice)
    a. Free range outdoors
    b. Fenced outdoors
    c. Free range indoors (housed)
    d. Caged indoors
    e. Free range outdoors during the day, housed at night
    f. Other (specify)

33. Do other animals at the farm have access to the areas where your chickens/hens are kept?
    a. Yes
    b. No
    c. There are no other animal species at my farm

34. If other animals at the farm have access to areas where your chickens/hens are kept, which animals? (multiple choice)
    a. Other poultry species
    b. Pigs
    c. Cattle
    d. Small ruminants
    e. Horses/donkeys
    f. Dogs
    g. Cats
    h. Rodents/rabbits
    i. Other (specify) ….

35. Do your chickens/hens mix with animals from **outside your own farm**?
    a. Yes, often
    b. Yes, sometimes
    c. Rarely
    d. Never

36. From where do you **most commonly** buy new chickens/hens? (single choice)
    a. Other farmers
    b. Local market
    c. Breeding company
    d. Breeding farms
    e. Other (specify) ….

<u>General farm management routines</u>

37. Which type(s) of feed to you give to your chickens/hens? (multiple choice)
    a. Pre-mix/commercial feed
    b. Feed mixed at farm
    c. Grains/crops grown at farm/locally grown
    d. Household/restaurant waste
    e. Scavenging
    f. Other (specify) ….

38. What type of feed do you **most commonly** give to your chickens/hens? (single choice)
    a. Pre-mix/commercial feed
    b. Feed mixed at farm
    c. Grains/crops grown at farm/locally grown
    d. Household/restaurant waste
    e. Scavenging
    f. Other (specify) ….

39. If you use pre-mix or commercial feed, does the feed contain already added medicines?

a. Yes
b. No
c. I don’t know
d. I never give pre-mix/commercial feed to my chickens/hens

40. If you use pre-mix or commercial feed that contains already added medicines, which medicines? (multiple choice)
    a. Antibiotics
    b. Vitamins
    c. Probiotics
    d. I don’t know
    e. Other (specify) …

41. Do you **usually** add any medicines to the feed yourself before giving it to your chickens/hens?
    a. Yes
    b. No

42. If you usually add medicines to the feed before giving it to your chickens/hens, which medicines? (multiple choice)
    a. Antibiotics
    b. Vitamins
    c. Probiotics
    d. I don’t know
    e. Other (specify) …

43. Do you sell eggs?
    a. Yes
    b. No

44. If you sell eggs, where do you **mostly** sell them? (single choice)
    a. To neighbors/friends/family
    b. To grocery store
    c. To company for packing/processing
    d. At local market
    e. To collectors
    f. Other (specify) ….

45. Do you sell live chickens/hens?
    a. Yes
    b. No

46. If you sell live chickens/hens, where do you **mostly** sell them? (single choice)
    a. To neighbors/friends/family
    b. To grocery store
    c. To company for slaughter
    d. At local market
    e. To collectors
    f. Other (specify) ….

47. Do you slaughter chickens/hens at the farm?

a. Yes
b. No

48. If you slaughter chickens/hens at the farm, do you have a specific area for slaughter that is separated from live animals?
    a. Yes
    b. No

49. Do you sell chicken/hen meat?
    a. Yes
    b. No

50. If you sell chicken/hen meat, where do you **mostly** sell it? (single choice)
    a. To neighbors/friends/family
    b. To grocery store
    c. At local market
    d. Other (specify) ….

51. How do you **usually** handle manure from your chickens/hens? (single choice)
    a. Do nothing
    b. Discard into environment
    c. Use or sell/give untreated as fertilizer
    d. Use or sell/give as fertilizer after treatment of the manure
    e. Use or sell/give as fertilizer after at least 1 month of composting
    f. Use for fuel (incl. biogas)
    g. Other (specify) ….

52. If you do any treatment of the manure before using/selling it as fertilizer, which treatment?
    a. I never treat the manure before using/selling it as fertilizer
    b. The treatment I do is: …

53. What do you **usually** do with chickens/hens that die from disease? (single choice)
    a. Throw in the trash
    b. Burn/destruct
    c. Use as animal feed
    d. Bury in the ground
    e. Use for household consumption
    f. Sell at local market
    g. Sell to other farmers as animal feed
    h. Leave on the ground
    i. Other (specify) ….

54. Do you usually empty the animal houses/areas between batches of chickens/hens (all-in/all-out system)?
    a. Yes
    b. No

55. If you empty the animal houses/areas between batches (all-in/all-out system), do you remove litter, manure and clean/disinfect animal houses before the next batch?
    a. Yes
    b. No

56. If you **do not** use an all-in/all-out system, how often do you remove litter, manure and clean/disinfect animal houses/areas where the chickens/hens are kept? (single choice)
    a. Once a week or more often
    b. Every second week
    c. Once a month
    d. More seldom

<u>Access to animal health services and veterinary drugs (incl. advice on how to use them)</u>

57. Do you have access to buying pharmaceuticals/veterinary drugs in your local community?
    a. Yes
    b. No

58. If you have access to buying pharmaceuticals/veterinary drugs, where do you **most commonly** buy them for your chickens/hens? (single choice)
    a. From other farmer
    b. At markets
    c. Via a governmental veterinarian, directly or at veterinary drug shop after prescription (a piece of paper with information about the needed medicine) from the veterinarian
    d. Via a private veterinarian, directly or at veterinary drug shop after prescription from the veterinarian
    e. At a veterinary drug shop (without prior prescription)
    f. From pharmaceutical company
    g. From a feed provider
    h. Other (specify) ….

59. If you buy from multiple sources, where do you **second most commonly** buy pharmaceuticals/veterinary drugs for your chickens/hens? (single choice)
    a. I don't buy from multiple sources
    b. From other farmer
    c. At markets
    d. Via a governmental veterinarian, directly or at veterinary drug shop after prescription (a piece of paper with information about the needed medicine) from the veterinarian
    e. Via a private veterinarian, directly or at veterinary drug shop after prescription from the veterinarian
    f. At a veterinary drug shop (without prior prescription)
    g. From pharmaceutical company
    h. From a feed provider
    i. Other (specify) ….

60. If you buy veterinary medicines at a veterinary drug shop, do you **usually** get a prescription from a veterinarian before you go and buy them?
    a. Yes
    b. No
    c. I don't buy medicines at a veterinary drug shop

61. If you buy medicines at a veterinary drug shop, does the person working there **usually** ask for a prescription before selling veterinary medicines to you?
    a. Yes

    b. No
    c. I don’t buy medicines at a veterinary drug shop

62. Is your farm a part of any farmers’ association?
    a. Yes
    b. No
    c. I don’t know

63. Do you have access to animal health service providers that give professional advice on how to handle and prevent diseases among your chickens/hens and help with diagnosis and treatments?
    a. Yes
    b. No
    c. I don’t know

64. If you have access to animal health service providers, which one(s)? (multiple choice)
    a. Governmental veterinarian
    b. Private veterinarian
    c. Veterinary drug shop worker (not veterinarian)
    d. Staff of drug company
    e. Other (specify) ….

65. If you have access to animal health services, do you use them for treatment of disease among your chickens/hens and/or advice on disease prevention?
    a. Yes, mostly
    b. Sometimes
    c. No

66. If you use animal health services for treatment and advice, which animal health service provider do you **most commonly** use? (single choice)
    a. Governmental veterinarian
    b. Private veterinarian
    c. Veterinary drug shop worker (not veterinarian)
    d. Staff of drug company
    e. Other (specify) ….

67. If you **do not** use the animal health services for treatment and advice, why? (multiple choice)
    a. Too expensive
    b. Not efficient/No added value
    c. Too complicated to get access
    d. Other (specify) ….

68. If you have access to animal health services, does the service include laboratory testing and/or autopsies when your chickens/hens are sick?
    a. Yes
    b. No
    c. I don’t know

69. If the animal health services include laboratory testing and/or autopsies, do you use these services?
    a. Yes, when needed

b. Sometimes
c. Never

70. If you do not use the service of laboratory testing and/or autopsies, why? (multiple choice)
    a. Too expensive
    b. Not efficient/Takes too long for results
    c. I don't know how to access them
    d. Other (specify) ….

71. Is your farm a part of any animal health program where you get routine monitoring and advice on the health of your chickens/hens?
    a. Yes
    b. No
    c. I don't know

72. Is your farm a part of any animal health program that provides vaccinations for your chickens/hens?
    a. Yes
    b. No
    c. I don't know

Disease issues at the farm

Information to the respondent: This section is for us to get a better understanding of which disease issues you deal with at your farm and if you have experienced situations where you have been unable to treat those diseases.

Common disease issues, history of treatment failure and record keeping

73. What have been the **most common** disease signs among your chickens/hens in the past 12 months (up to three options can be chosen)?
    a. Respiratory (problems with breathing/coughing/nasal discharge)
    b. Swollen/red/runny eyes
    c. Digestive/intestinal
    d. Skin disease/wounds
    e. External parasites
    f. Lameness
    g. Neurological
    h. Fatigue
    i. Weight loss
    j. Anorexia
    k. Sudden death
    l. No disease issues the past 12 months
    m. Other (specify) ….

74. Have you ever experienced situations where medicines did not work when you tried to treat sick chickens/hens?
    a. Yes
    b. No

75. If you have experienced that medicines did not work, which disease signs have your birds **most commonly** shown in those cases? (single choice)
    a. Respiratory (problems with breathing/coughing/nasal discharge)
    b. Swollen/red/runny eyes
    c. Digestive/intestinal
    d. Skin disease/wounds
    e. External parasites
    f. Lameness
    g. Neurological
    h. Fatigue
    i. Weight loss
    j. Anorexia
    k. Other (specify) ….

76. If you have experienced that medicines did not work, which medicine(s)? (Free text)

77. Do you keep records of disease and mortality among your chickens/hens?
    a. Yes
    b. No

Disease prevention, disease management and treatment routines

Information to the respondent: This section includes questions about what you do in order to prevent your animals from becoming sick and what you do when/if they do get sick. The disease management section includes routines for diagnosing disease, treatment with veterinary drugs and handling of leftover and/or expired drugs.

78. To prevent your chickens/hens from becoming sick, do you:
    a. Fence them? (Yes/No)
    b. **Usually** isolate/quarantine newly bought animals for some time before mixing them with the chickens/hens that are already at the farm? (Yes/No)
    c. Give them antibiotics? (Yes/No)
    d. Give them feed that is supplemented with antibiotics? (Yes/No)
    e. Vaccinate them against any diseases? (Yes/No)

79. Against which disease(s) do you vaccinate your chickens/hens? (multiple choice)
    a. Newcastle disease
    b. Gumboro disease
    c. Marek's disease
    d. Avian influenza
    e. Pasteurellosis (Fowl cholera)
    f. Other (specify)

80. Do you:
    a. Wash your hands before entering the areas where your animals are kept? (Yes/No)
    b. Wash your hands after visiting the areas where your animals are kept? (Yes/No)
    c. Have separate footwear (e.g. gum boots) or plastic boot covers that you use only in the areas where your chickens/hens are kept? (Yes/No)

81. Do you give your chickens/hens antibiotics to make them grow faster and/or better?
    a. Yes
    b. No

82. Do you give your hens antibiotics to make them lay more eggs?
    a. Yes
    b. No
    c. I don't keep layer hens

83. Who will **usually** diagnose disease among the chickens/hens at the farm? (single choice)
    a. Myself
    b. Governmental veterinarian
    c. Private veterinarian
    d. Veterinary drug shop worker (not veterinarian)
    e. Human doctor
    f. Other farmer
    g. Friend/family member
    h. Other (specify) ….

84. What do you **usually** do first when the chickens/hens at your farm get sick? (single choice)
    a. Nothing
    b. Give them medicine(s) from a veterinary drug shop/market
    c. Give them traditional medicine/vitamins/herbs
    d. Give them medicine(s) that was left by a veterinarian at a previous visit
    e. Consult a governmental veterinarian
    f. Consult a private veterinarian
    g. Other (specify) ….

85. Do you **usually** isolate chickens/hens that become sick from the rest of the poultry in the flock?
    a. Yes
    b. No

86. When you use antibiotics to treat disease among your chickens/hens, which animals do you **usually** treat? (single choice)
    a. All poultry at the farm
    b. All chickens/hens at the farm
    c. Only the chickens/hens that are sick
    d. All chickens/hens that are sick and all animals in contact with the sick chickens/hens
    e. All animals at the farm
    f. I never use antibiotics to treat my chickens/hens

87. From where do you **usually** get advice on **when** to use antibiotics for your chickens/hens? (single choice)
    a. I don't get advice, I use my own judgement
    b. From other farmers
    c. From veterinary drug shop worker (not veterinarian)
    d. From package/label of the medicine
    e. From a governmental veterinarian
    f. From a private veterinarian

g. From market sales person
h. From human doctor
i. From feed provider
j. From friends/family
k. I never use antibiotics to treat my chickens/hens
l. Other (specify) ….

88. When you use antibiotics to treat disease among your chickens/hens, for how long do you **usually** treat them? (single choice)
    a. Until animal(s) cured
    b. Until animal(s) begin to recover
    c. As advised by a governmental veterinarian
    d. As advised by a private veterinarian
    e. As advised by other (e.g. sales person, other farmer, family/friends, human doctor)
    f. As instructed on the package/label of the medicine
    g. Until package is empty
    h. One treatment only
    i. I never use antibiotics to treat my chickens/hens
    j. Other (specify) ….

89. When treating your chickens/hens with antibiotics, whose instructions do you **usually** follow on **how** to use them (dose, treatment length, administration route etc.)? (single choice)
    a. I don't get instructions, I use my own judgement
    b. Other farmers'
    c. A veterinary drug shop worker's (not veterinarian)
    d. The instructions on the package/label of the medicine
    e. A governmental veterinarian's
    f. A private veterinarian's
    g. A market sales person's
    h. A human doctor's
    i. A feed provider's
    j. Friends'/family's
    k. I never use antibiotics to treat my chickens/hens
    l. Other's (specify) ….

90. When you use antibiotics to treat disease among chickens/hens, who **usually** administers the drug? (single choice)
    a. Myself, by own experience
    b. Myself, after instructions from a governmental veterinarian
    c. Myself, after instructions from a private veterinarian
    d. Governmental veterinarian
    e. Private veterinarian
    f. I never use antibiotics to treat my chickens/hens
    g. Other (specify) ….

91. Do you ever give a **higher** dose of antibiotics than the recommended to your chickens/hens?
    a. Yes
    b. No
    c. I never use antibiotics to treat my chickens/hens

92. Do you ever give a **lower** dose of antibiotics than the recommended to your chickens/hens?

a. Yes
b. No
c. I never use antibiotics to treat my chickens/hens

93. Do you ever stop giving your chickens/hens antibiotics earlier than recommended if they seem healthy?
    a. Yes
    b. No
    c. I never use antibiotics to treat my chickens/hens

94. If the antibiotic treatment of sick chickens/hens is not effective or does not work, what do you **usually** do? (single choice)
    a. Increase the dose
    b. Switch to another antibiotic or combine the ongoing treatment with another antibiotic
    c. Switch to other type of medicine
    d. Switch to herbal/traditional medicine
    e. Go back to the veterinary drug shop for advice (from non-veterinarian)
    f. Contact governmental veterinarian
    g. Contact private veterinarian
    h. Contact other person (not veterinarian) for advice
    i. Slaughter the sick animal(s) for meat
    j. Euthanize the sick animal(s)
    k. Nothing
    l. I have never experienced that antibiotic treatment is not effective or does not work
    m. I never use antibiotics to treat my chickens/hens
    n. Other (specify) ….

95. What do you **usually** do with expired/leftover veterinary antibiotics? (single choice)
    a. Throw in the trash/latrine
    b. Keep for later use
    c. Give to other farmer
    d. Leave to pharmacy/veterinary drug shop
    e. I never use antibiotics on my farm
    f. Other (specify) ….

96. Does it happen that you give human medicines to your chickens/hens when they become sick?
    a. Yes, often
    b. Sometimes
    c. No

97. Do you keep records of the use of medicines for the chickens/hens at your farm (e.g. treatment dates, name of medicine, dose)?
    a. Yes
    b. No

Knowledge about antibiotics and antibiotic use

Information to the respondent: This is the final section of the questionnaire. You will be asked a few questions and presented with some true or false statements about antibiotics and resistance to

antibiotics. Some questions might seem similar, but take your time to listen to the questions carefully and have them repeated if you do not fully understand the first time.

98. What are antibiotics **supposed** to be used for? (single choice)
    a. Prevent animals from becoming sick
    b. Treat sick animals
    c. Make animals grow faster/better
    d. Prevent animals from becoming sick <u>and</u> make animals grow faster/better
    e. Prevent animals from becoming sick <u>and</u> treat sick animals
    f. Treat sick animals <u>and</u> make animals grow faster/better
    g. Prevent animals from becoming sick, treat sick animals <u>and</u> to make animals grow faster/better

Instruction to the enumerator:

The option "Cannot answer" in the True/False section shall not be read to the respondent. Tick this option if the respondent really have no idea about what to answer. The respondent is not allowed to go back and change an already given answer. Please read the questions again if the respondent doesn't understand the first time.

99. Do you think that the following statements are true or false?
    a. Antibiotics can treat all kinds of diseases (True/False/Cannot answer)

    b. Antibiotics can treat diseases caused by viruses (True/False/Cannot answer)

    c. Antibiotics can treat diseases caused by bacteria (True/False/Cannot answer)

    d. Antibiotics are the same as anti-inflammatory drugs (True/False/Cannot answer)

    e. Different types of antibiotics are needed for different diseases (True/False/Cannot answer)

    f. As a general rule, you should stop treatment with antibiotics when the animal's condition starts to improve (True/False/Cannot answer)

    g. Using antibiotics too often can make diseases difficult to treat in the future (True/False/Cannot answer)

    h. Animals can become resistant to antibiotics if antibiotics are used in the wrong way/too often (True/False/Cannot answer)

    i. Bacteria that cause disease can become resistant to antibiotics if used in the wrong way/too often (True/False/Cannot answer)

    j. Viruses that cause disease can become resistant to antibiotics if used in the wrong way/too often (True/False/Cannot answer)

    k. Resistance against antibiotics can make it more difficult to succeed with antibiotic treatment in animals when they get sick (True/False/Cannot answer)

    l. Bacteria resistant to antibiotics can spread from one animal to another (True/False/Cannot answer)

m. Bacteria resistant to antibiotics can spread between animals and humans (True/False/Cannot answer)

n. Bacteria resistant to antibiotics can spread from animals to humans through animal source foods, e.g. meat (True/False/Cannot answer)

o. Bacteria resistant to antibiotics can spread through manure from animals (True/False/Cannot answer)

p. Using too much antibiotics in animals can make it more difficult to treat some diseases in humans (True/False/Cannot answer)

q. Antibiotic resistance in human bacteria is only linked to the use of antibiotics in humans and not in animals (True/False/Cannot answer)

Information to the respondent: To be able to contact you if we would need you to clarify any answer given in the survey, we would like to ask you for your phone number.

100. Respondent's phone number

Final words to the respondent: Thank you for taking the time to answer the questions in this questionnaire and for letting us visit your farm. Your participation is truly appreciated. Kind regards from the research team.

101. Latitude …
102. Longitude …
