# Supplementary material for: Understanding Vietnamese chicken farmers’ knowledge and practices related to antimicrobial resistance using an item response theory approach
Source: Front Vet Sci. 2024 Apr 5;11:1319933. doi: 10.3389/fvets.2024.1319933 (PMC11027563; doi:10.3389/fvets.2024.1319933)
Supplement: Supplementary file 2 [file Data_Sheet_2.pdf]

Supplementary Material S2

1 Supplementary Figures S1A-C

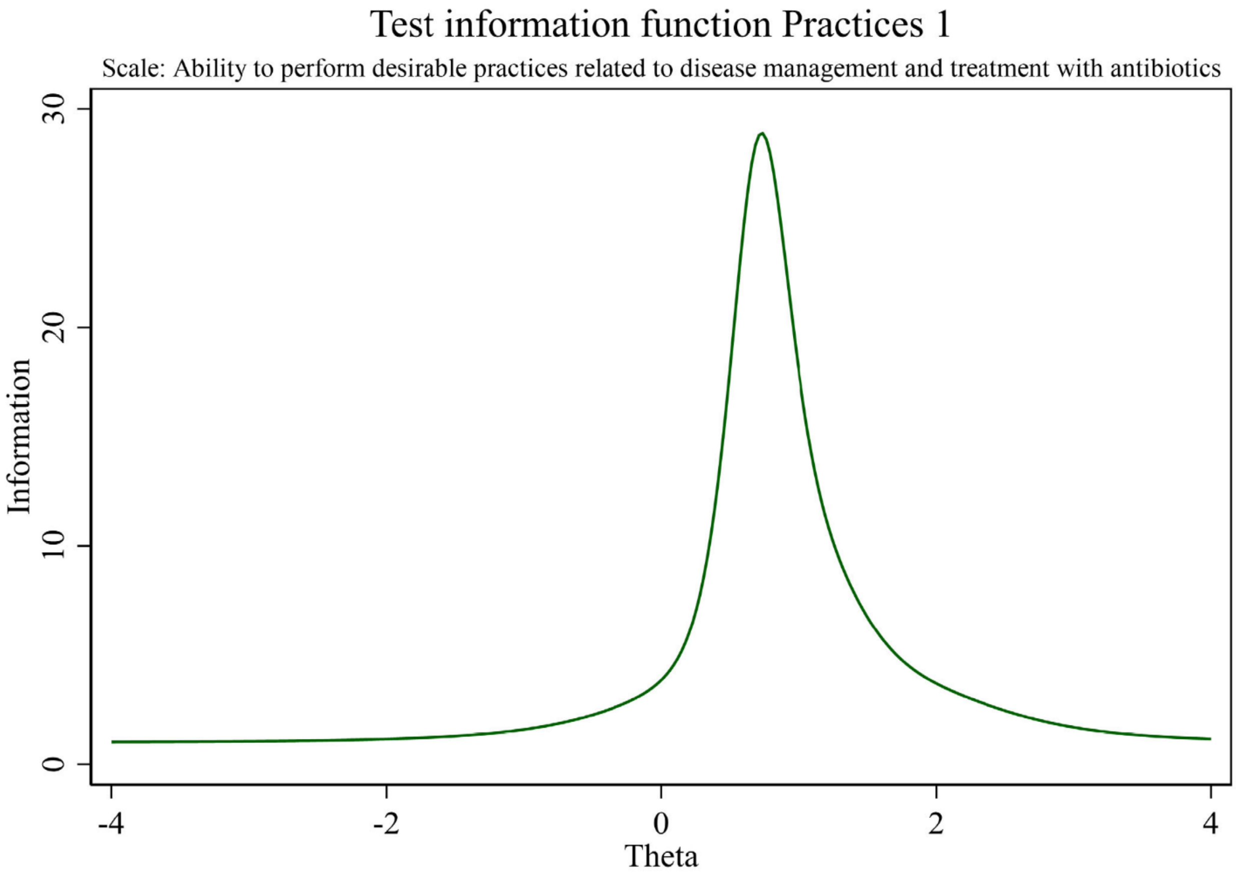

Figure S1A. Test information function of the Practices 1 scale based on responses in a questionnaire distributed among small- and medium-scale chicken farmers in Thai Nguyen City, Dong Hy and Vo Nhai districts in Vietnam. Practices 1 = Ability to perform desirable practices related to disease management and treatment with antibiotics.

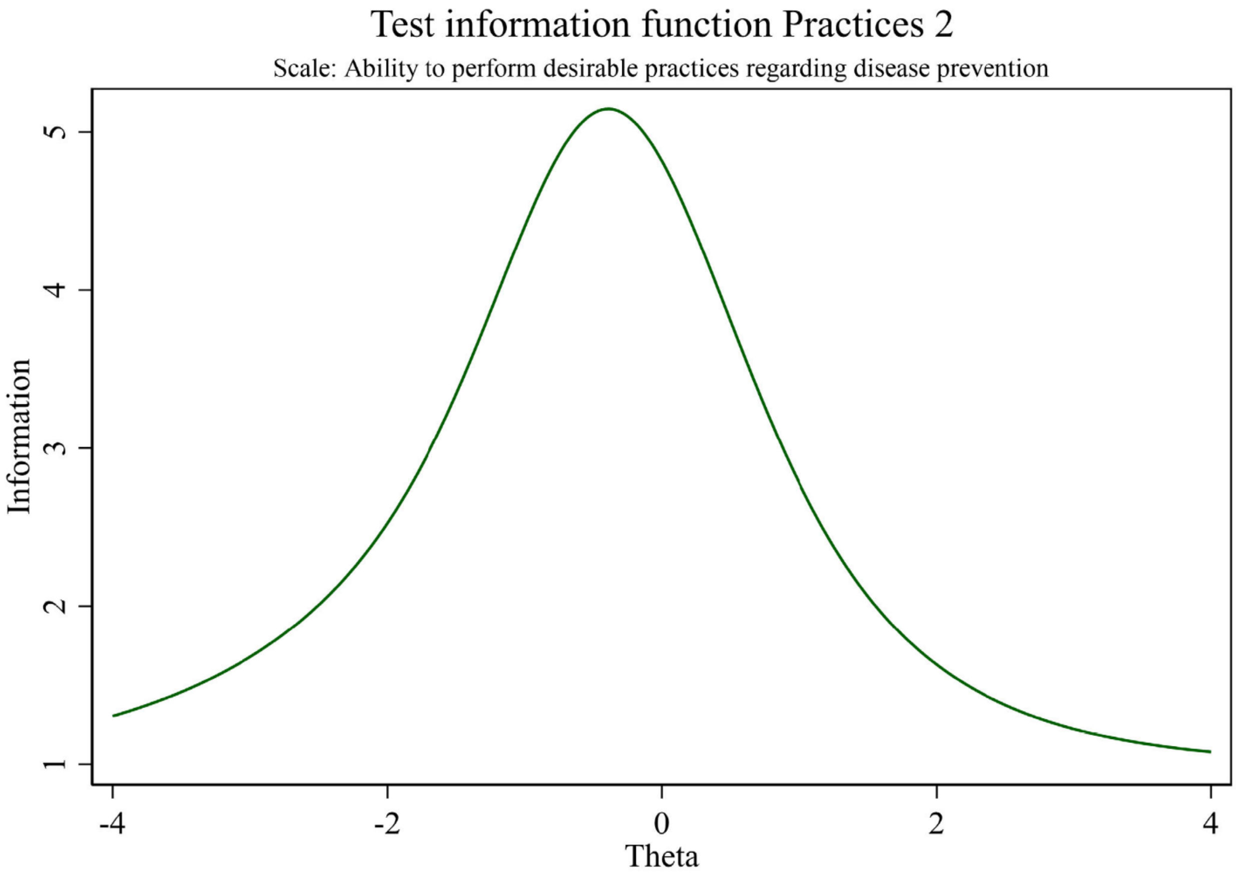

Figure S1B. Test information function of the Practices 2 scale based on responses in a questionnaire distributed among small- and medium-scale chicken farmers in Thai Nguyen City, Dong Hy and Vo Nhai districts in Vietnam. Practices 2 = Ability to perform desirable practices regarding disease prevention.

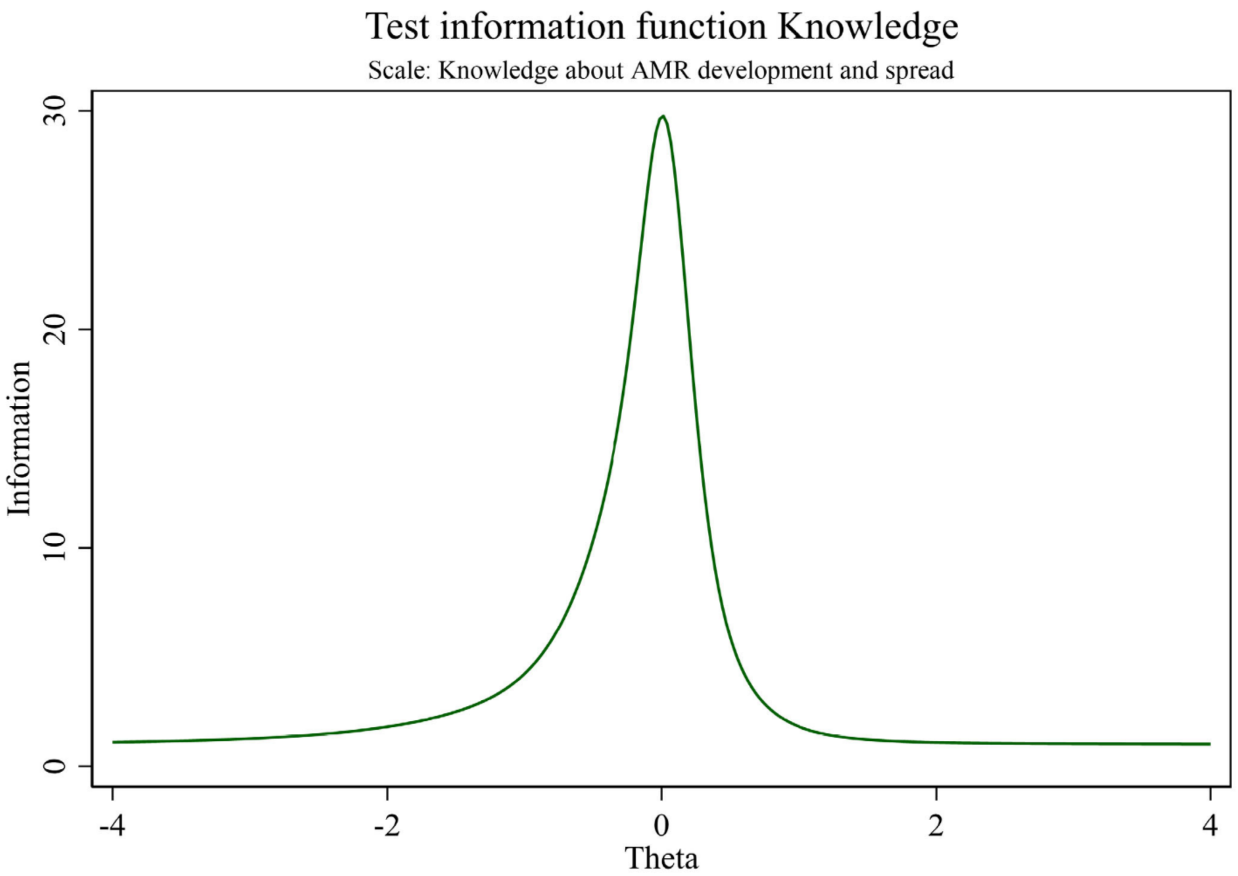

Figure S1C. Test information function of the Knowledge scale based on responses in a questionnaire distributed among small- and medium-scale chicken farmers in Thai Nguyen City, Dong Hy and Vo Nhai districts in Vietnam. Knowledge = Knowledge about antimicrobial resistance (AMR) development and spread.

2 Supplementary Tables

2.1 Tables S1-S10

Table S1. Supplementary demographics and farm characteristics among small- and medium-scale chicken farmers in Thai Nguyen City, Dong Hy and Vo Nhai districts in Vietnam (n=305).

Item	Option	% (number)
District	Dong Hy	18.4 (56)
	Thai Nguyen	16.2 (51)
	Vo Nhai	64.9 (198)
Age category of respondent (yrs)	21-30	3.6 (11)
	31-40	17.4 (53)
	41-50	24.9 (76)
	51-60	34.4 (105)
	>60	19.3 (59)
	Unknown	0.3 (1)
Median age (yrs)		53
Main role of respondent	Household head	65.3 (199)
	Wife/husband	29.5 (90)
	Child	3.6 (11)
	Parents	0.7 (2)
	Grandparents	0 (0)
	Worker	0 (0)
	Other	1.0 (3)
Years of farming experience	1-10	22.3 (68)
	11-20	34.8 (106)
	21-30	27.2 (83)
	31-40	13.1 (40)
	>40	2.6 (8)
Median experience (yrs)		20
Median number of chickens/hens (heads)	70	
How many people (other than the farmer) work with the chickens/hens?	0	22.0 (67)
	1	53.1 (162)
	2	20.0 (61)
	3	3.0 (9)
	4	1.3 (4)
	5	0.3 (1)
	6	0.3 (1)
Are there hired workers on the farm?	No	100 (305)
Animal species kept	Dual purpose chickens/hens	56.1 (171)
	Broiler chickens (not dual purpose)	71.0 (215)
	Layer hens (not dual purpose)	42.3 (126)
	Other poultry	41.0 (125)
	Pigs	34.4 (105)
	Cattle	26.9 (82)
	Small ruminants	1.3 (4)
	Donkeys/horses	0 (0)

	Dogs	92.8 (283)
	Cats	46.2 (141)
	Rodents/rabbits	0.3 (1)
	Other species	0.3 (1)
Farms that keep at least one other species than chickens/hens		98.4 (300)

Table S2. Buying, selling and slaughtering practices among small- and medium-scale chicken farmers in Thai Nguyen City, Dong Hy and Vo Nhai districts in Vietnam.

**Item**	**Option**	**% (number)**
From where do you most commonly buy new chickens/hens? (n=305)	Other farmers	17.4 (53)
	Local market	18.0 (55)
	Breeding company	6.2 (19)
	Breeding farms	30.8 (94)
	Other	27.5 (84)
Do you sell live chickens/hens? (n=305)	Yes	64.3 (196)
If you sell live chickens/hens, where do you mostly sell them? (n=196)	To neighbors/friends/family	71.4 (140)
	To grocery store	0.5 (1)
	To company for slaughter	0.5 (1)
	At local market	15.3 (30)
	To collectors	11.7 (23)
	Other	0.5 (1)
Do you sell eggs? (n=305)	Yes	37.0 (113)
If you sell eggs, where do you mostly sell them? (n=113)	To neighbors/friends/family	81.4 (92)
	To grocery store	2.7 (3)
	To company for packing/processing	0 (0)
	At local market	15.0 (17)
	To collectors	0.8 (1)
	Other	0 (0)
Do you slaughter chickens/hens at the farm? (n=305)	Yes	42.3 (129)
Do you sell chicken/hen meat? (n=305)	Yes	21.0 (64)
If you sell chicken/hen meat, where do you mostly sell it? (n=64)	To neighbors/friends/family	78.1 (50)
	To grocery store	1.6 (1)
	At local market	17.2 (11)
	Other	3.1 (2)

Table S3. Chicken housing practices among small- and medium-scale chicken farmers in Thai Nguyen City, Dong Hy and Vo Nhai districts in Vietnam.

**Item**	**Option**	**% (number)**
How do you keep your dual purpose chickens/hens? (n=171)	Free range outdoors	17.5 (30)
	Fenced outdoors	50.3 (86)
	Free range indoors (housed)	5.9 (10)
	Caged indoors	0.6 (1)
	Free range outdoors during the day, housed at night	25.7 (44)
	Other	0 (0)
How do you keep your broiler chickens? (n=215)	Free range outdoors	17.7 (38)

	Fenced outdoors	40.5 (87)
	Free range indoors (housed)	8.8 (19)
	Caged indoors	1.9 (4)
	Free range outdoors during the day, housed at night	31.2 (67)
	Other	0 (0)
How do you keep your layer hens? (n=126)	Free range outdoors	16.7 (21)
	Fenced outdoors	38.9 (49)
	Free range indoors (housed)	3.2 (4)
	Caged indoors	1.6 (2)
	Free range outdoors during the day, housed at night	39.7 (50)
	Other	0 (0)
Do other animals at the farm have access to the areas where your chickens/hens are kept? (n=305)	Yes	68.2 (208)
	No	27.5 (84)
	There are no other animal species at my farm	4.3 (13)
If other animals at the farm have access to the areas where your chickens/hens are kept, which species? (n=208/305)	Other poultry	36.1/24.6 (75)
	Pigs	3.9/2.6 (8)
	Cattle	8.2 /5.6 (17)
	Small ruminants	0.5/0.3 (1)
	Donkeys/horses	0 (0)
	Dogs	85.6/58.4 (178)
	Cats	36.1/24.6 (75)
	Rodents/rabbits	0.5/0.3 (1)
	Other	0.5/0.3 (1)
Do your chickens/hens mix with animals from outside your own farm? (n=305)	Yes, often	3.3 (10)
	Yes, sometimes	7.5 (23)
	Rarely	6.6 (20)
	Never	82.6 (252)
If you slaughter chickens/hens at the farm, do you have a specific area for slaughter that is separated from live animals? (n=129)	Yes	85.3 (110)

Table S4. Chicken feeding and manure management at small- and medium-scale chicken farms in Thai Nguyen City, Dong Hy and Vo Nhai districts in Vietnam.

Item	Option	% (number)
Which type(s) of feed to you give to your chickens/hens? (multiple choice) (n=305)	Pre-mix/commercial feed	38.7 (118)
	Feed mixed at farm	14.1 (43)
	Grains/crops grown at farm/locally grown	96.7 (295)
	Household/restaurant waste	7.9 (24)
	Scavenging	3.9 (12)
What type of feed do you most commonly give to your chickens/hens? (n=305)	Pre-mix/commercial feed	3.0 (9)
	Feed mixed at farm	3.6 (11)
	Grains/crops grown at farm/locally grown	92.8 (283)
	Household/restaurant waste	0.7 (2)
	Scavenging	0 (0)

If you use pre-mix/commercial feed, does the feed contain already added medicines? (n=305)	Yes	10.8 (33)
	No	23.0 (70)
	I don't know	23.6 (72)
	I never give pre-mix/commercial feed	42.6 (130)
If the pre-mix/commercial feed contains medicines, which medicines? (multiple choice) (n=33)	Antibiotics	51.5 (17)
	Vitamins	69.7 (23)
	Probiotics	42.4 (14)
	I don't know	9.1 (3)
	Other	0 (0)
Do you usually add any medicines to the feed before giving it to your chickens/hens? (n=305)	Yes	24.3 (74)
If you usually add medicines to the feed, which medicines? (n=74/305)	Antibiotics	70.3/17.1 (52)
	Vitamins	70.3/17.1 (52)
	Probiotics	56.8/13.8 (42)
	I don't know	2.7/0.7 (2)
How do you usually handle manure from your chickens/hens? (n=305)	Do nothing	14.1 (43)
	Discard into the environment	9.2 (28)
	Use or sell/give untreated as fertilizer	30.8 (94)
	Use or sell/give after treatment of the manure	39.0 (119)
	Use or sell/give after at least 1 month of composting	4.9 (15)
	Use for fuel (incl. biogas)	0.7 (2)
	Other	1.3 (4)

Table S5. Handling of diseased animals and cleaning routines at small- and medium-scale chicken farms in Thai Nguyen City, Dong Hy and Vo Nhai districts in Vietnam.

**Item**	**Option**	**% (number)**
What do you usually do with chickens/hens that die from disease? (n=305)	Throw in the trash	12.1 (37)
	Burn/destruct	12.8 (39)
	Use as animal feed	4.9 (15)
	Bury in the ground	65.6 (200)
	Use for household consumption	2.6 (8)
	Sell at local market	0 (0)
	Sell to other farmers as animal feed	0 (0)
	Leave on the ground	0.3 (1)
	Other	1.6 (5)
Do you usually empty the animal houses/areas between batches of chickens/hens (all-in/all-out system)? (n=305)	Yes	15.1 (46)
If you use all-in/all-out system, do you remove litter, manure and clean/disinfect animal houses before next batch? (n=46)	Yes	93.5 (43)
If you do not use an all-in/all-out system, how often do you remove litter, manure and clean/disinfect animal houses/areas where the chickens/hens are kept? (n=259)	Once a week or more often	4.6 (12)

	Every second week	9.7 (25)
	Once a month	51.4 (133)
	More seldom	34.4 (89)

Table S6. Disease issues at small- and medium-scale chicken farms in Thai Nguyen City, Dong Hy and Vo Nhai districts in Vietnam.

Item	Option	% (number)
What have been the most common disease signs among your chickens/hens in the past 12 months? (up to three options can be chosen) (n=305)	Respiratory	34.8 (106)
	Swollen/red/runny eyes	2.3 (7)
	Digestive/intestinal	60.0 (183)
	Skin disease/wounds	1.0 (3)
	External parasites	1.6 (5)
	Lameness	4.3 (13)
	Neurological	0.3 (1)
	Fatigue	9.5 (29)
	Weight loss	1.3 (4)
	Anorexia	2 (6)
	Sudden death	7.2 (22)
	Other	0.7 (2)
	No disease issues the past 12 months	26.2 (80)
Have you ever experienced situations where medicines did not work when you tried to treat sick chickens/hens? (n=305)	Yes	28.2 (86)
If you have experienced that medicines did not work, which disease signs have your birds most commonly shown in those cases? (n=87)	Respiratory	33.3 (29)
	Swollen/red/runny eyes	0 (0)
	Digestive/intestinal	48.3 (42)
	Skin disease/wounds	0 (0)
	External parasites	1.2 (1)
	Lameness	5.8 (5)
	Neurological	1.2 (1)
	Fatigue	4.6 (4)
	Weight loss	4.6 (4)
	Anorexia	0 (0)
	Other	1.2 (1)
Do you keep records of disease and mortality among your chickens/hens? (n=305)	Yes	1.0 (3)
Against which disease(s) do you vaccinate your chickens/hens? (multiple choice) (n=165/305)	Newcastle disease	86.7/46.9 (143)
	Gumboro disease	44.2/23.9 (73)
	Marek’s disease	4.9/2.6 (8)
	Avian influenza	21.8/11.8 (36)
	Pasteurellosis (Fowl cholera)	50.3/27.2 (83)

Table S7. Practices related to antimicrobial resistance (AMR) development and spread, including disease prevention (not provided in tables S3-S6), among small- and medium-scale chicken farmers in Thai Nguyen City, Dong Hy and Vo Nhai districts in Vietnam.

Item	Option	% (number)
If you have access to buying pharmaceuticals/veterinary drugs, where do you most commonly buy them for your chickens/hens? (n=294)	At veterinary drug shop without prior prescription	86.7 (255)
	Via a governmental veterinarian, directly or via prescription	4.4 (13)
	From pharmaceutical company	2.0 (6)
	From a feed provider	2.0 (6)
	Via a private veterinarian, directly or via prescription	2.0 (6)
	At markets	1.4 (4)
	Other	1.0 (3)
	From other farmer	0.3 (1)
If you have access to animal health services, do you use them for treatment of disease among your chickens/hens and/or advice on disease prevention? (n=97)	Yes, mostly	25.8 (25)
	Sometimes	61.9 (60)
	No	12.4 (12)
If you use animal health services for treatment and advice, which animal health service provider do you most commonly use? (n=85)	Veterinary drug shop worker (not veterinarian)	54.1 (46)
	Governmental veterinarian	30.6 (26)
	Private veterinarian	11.8 (10)
	Staff of drug company	2.4 (2)
	Other	1.2 (1)
If the animal health services include laboratory testing and/or autopsies, do you use these services? (n=31)	Yes, when needed	58.1 (18)
	Sometimes	16.1 (5)
	Never	25.8 (8)
To prevent your chickens/hens from becoming sick, do you (multiple choice): (n=305)	Give them antibiotics	65.3 (199)
	Fence them	63.0 (192)
	Usually isolate/quarantine newly bought animals for some time	57.4 (175)
	Vaccinate	54.1 (165)
	Give them feed that is supplemented with antibiotics	32.1 (98)
Do you (multiple choice): (n=305)	Wash your hands after visiting the areas where your animals are kept?	92.5 (282)
	Wash your hands before entering the areas where your animals are kept?	23.6 (72)
	Have separate footwear (e.g. gum boots) or plastic boot covers that you use only in the areas where your chickens/hens are kept?	41.3 (126)
Do you give your chickens/hens antibiotics to make them grow faster and/or better? (n=305)	Yes	2.0 (6)
Do you give your hens antibiotics to make them lay more eggs? (n=225)	Yes	0.9 (2)

Who will usually diagnose disease among the chickens/hens at the farm? (n=305)	Myself	52.1 (159)
	Veterinary drug shop worker (not veterinarian)	31.8 (97)
	Private veterinarian	7.5 (23)
	Governmental veterinarian	5.9 (18)
	Friend/family member	1.3 (4)
	Other farmer	0.7 (2)
	Human doctor	0 (0)
	Other	0.7 (2)
What do you usually do first when the chickens/hens at your farm get sick? (n=305)	Give them medicine(s) from a veterinary drug shop/market	47.2 (144)
	Consult a private veterinarian	32.1 (98)
	Consult a governmental veterinarian	7.9 (24)
	Nothing	4.3 (13)
	Give them traditional medicine/vitamins/herbs	2.0 (6)
	Give them medicine(s) that was left by a veterinarian at a previous visit	1.6 (5)
	Other	4.9 (15)
Do you usually isolate chickens/hens that become sick from the rest of the poultry in the flock? (n=305)	Yes	80.0 (244)
When you use antibiotics to treat disease among your chickens/hens, which animals do you usually treat? (n=305)	All chickens/hens at the farm	45.6 (139)
	Only the chickens/hens that are sick	42.3 (129)
	All poultry at the farm	5.9 (18)
	All chickens/hens that are sick and all animals in contact with the sick chickens/hens	0.7 (2)
	All animals at the farm	0 (0)
From where do you usually get advice on when to use antibiotics for your chickens/hens? (n=305)	From veterinary drug shop worker (not veterinarian)	43.6 (133)
	I don't get advice, I use my own judgement	24.6 (75)
	From a private veterinarian	14.4 (44)
	From a governmental veterinarian	7.2 (22)
	From package/label of the medicine	2.3 (7)
	From other farmers	0.7 (2)
	From feed provider	0.7 (2)
	From friends/family	0.7 (2)
	From market sales person	0.3 (1)
	From human doctor	0 (0)
When you use antibiotics to treat disease among your chickens/hens, for how long do you usually treat them? (n=305)	Until animal(s) cured	48.5 (148)
	As advised by a private veterinarian	22.6 (69)
	Until animal(s) begin to recover	8.5 (26)
	As advised by a governmental veterinarian	6.9 (21)
	As instructed on the package/label of the medicine	6.2 (19)
	As advised by other (e.g. sales person, other farmer, family/friends, human doctor)	1.0 (3)
	Until package is empty	0 (0)
	One treatment only	0 (0)

When treating your chickens/hens with antibiotics, whose instructions do you usually follow on how to use them (dose, treatment length, administration route etc.)? (n=305)	A veterinary drug shop worker's (not veterinarian)	42.3 (129)
	I don't get advice, I use my own judgement	19.7 (60)
	A private veterinarian's	17.4 (53)
	A governmental veterinarian's	6.6 (20)
	The instructions on the package/label of the medicine	5.3 (16)
	A feed provider's	1.3 (4)
	Friends'/family's	1.0 (3)
	Other farmers'	0.3 (1)
	A market sales person's	0 (0)
	A human doctor's	0 (0)
When you use antibiotics to treat disease among chickens/hens, who usually administers the drug? (n=305)	Myself, by own experience	60.0 (183)
	Myself, after instructions from a private veterinarian	19.0 (58)
	Private veterinarian	8.9 (27)
	Myself, after instructions from a governmental veterinarian	4.9 (15)
	Governmental veterinarian	0.7 (2)
Do you ever give a higher dose of antibiotics than the recommended to your chickens/hens? (n=305)	Yes	19.3 (59)
Do you ever give a lower dose of antibiotics than the recommended to your chickens/hens? (n=305)	Yes	1.3 (4)
Do you ever stop giving your chickens/hens antibiotics earlier than recommended if they seem healthy? (n=305)	Yes	15.1 (46)
Does it happen that you give human medicines to your chickens/hens when they become sick? (n=305)	Often or Sometimes	35.1 (107)
If the antibiotic treatment of sick chickens/hens is not effective or does not work, what do you usually do? (n=305)	Switch to other type of medicine	24.9 (76)
	Go back to the veterinary drug shop for advice (from non-veterinarian)	21.0 (64)
	Contact private veterinarian	13.4 (41)
	Contact governmental veterinarian	7.9 (24)
	Increase the dose	7.5 (23)
	Switch to another antibiotic or combine the ongoing treatment with another antibiotic	7.2 (22)
	Contact other person (not veterinarian) for advice	2.3 (7)
	Slaughter the sick animal(s) for meat	2.3 (7)
	Switch to herbal/traditional medicine	0.7 (0)
	Nothing	0.7 (2)
	Euthanize the sick animal(s)	0 (0)
	I have never experienced that antibiotic treatment is not effective or does not work	3.9 (12)
What do you usually do with expired/leftover veterinary antibiotics? (n=305)	Throw in the trash/latrine	77.1 (235)
	Keep for later use	14.1 (43)
	Give to other farmer	0.7 (2)
	Leave to pharmacy/veterinary drug shop	0 (0)

Do you keep records of the use of medicines for the chickens/hens at your farm (e.g. treatment dates, name of medicine, dose)? (n=305)	Yes	0.7 (2)

Table S8. Knowledge about antibiotics and development and spread of AMR among small- and medium-scale chicken farmers in Thai Nguyen City, Dong Hy and Vo Nhai districts in Vietnam.

**Item**	**Option**	**% (number)**
What are antibiotics supposed to be used for? (n=305)	Treat sick animals	68.9 (210)
	Prevent animals from becoming sick and treat sick animals	17.4 (53)
	Prevent animals from becoming sick, treat sick animals and to make animals grow faster/better	7.2 (22)
	Prevent animals from becoming sick	4.6 (14)
	Treat sick animals and make animals grow faster/better	1.0 (3)
	Prevent animals from becoming sick and make animals grow faster/better	0.7 (2)
	Make animals grow faster/better	0.3 (1)
Antibiotics can treat all kinds of diseases (n=305)	True	44.3 (135)
	False	53.8 (164)
	Cannot answer	2.0 (6)
Antibiotics can treat diseases caused by viruses (n=305)	True	40.7 (124)
	False	50.8 (155)
	Cannot answer	8.5 (26)
Antibiotics can treat diseases caused by bacteria (n=304)	True	87.2 (265)
	False	6.9 (21)
	Cannot answer	5.9 (18)
Antibiotics are the same as anti-inflammatory drugs (n=304)	True	50.7 (154)
	False	38.8 (118)
	Cannot answer	10.5 (32)
Different types of antibiotics are needed for different diseases (n=303)	True	80.2 (243)
	False	17.5 (53)
	Cannot answer	2.3 (7)
As a general rule, you should stop treatment with antibiotics when the animal's condition starts to improve (n=303)	True	43.9 (133)
	False	50.2 (152)
	Cannot answer	5.9 (18)
Using antibiotics too often can make diseases difficult to treat in the future (n=304)	True	70.2 (214)
	False	20.4 (62)
	Cannot answer	9.2 (28)
Animals can become resistant to antibiotics if antibiotics are used in the wrong way/too often (n=304)	True	66.1 (201)
	False	20.7 (63)
	Cannot answer	13.2 (40)

Bacteria that cause disease can become resistant to antibiotics if used in the wrong way/too often (n=303)	True	72.9 (221)
	False	14.9 (45)
	Cannot answer	12.2 (37)
Viruses that cause disease can become resistant to antibiotics if used in the wrong way/too often (n=303)	True	48.8 (148)
	False	32.3 (98)
	Cannot answer	18.8 (57)
Resistance against antibiotics can make it more difficult to succeed with antibiotic treatment in animals when they get sick (n=303)	True	76.9 (233)
	False	10.9 (33)
	Cannot answer	12.2 (37)
Bacteria resistant to antibiotics can spread from one animal to another (n=304)	True	69.4 (211)
	False	15.8 (48)
	Cannot answer	14.8 (45)
Bacteria resistant to antibiotics can spread between animals and humans (n=302)	True	51.7 (156)
	False	32.5 (98)
	Cannot answer	15.9 (48)
Bacteria resistant to antibiotics can spread from animals to humans through animal source foods, e.g. meat (n=302)	True	45.0 (136)
	False	37.4 (113)
	Cannot answer	17.6 (53)
Bacteria resistant to antibiotics can spread through manure from animals (n=302)	True	52.0 (157)
	False	33.4 (101)
	Cannot answer	14.6 (44)
Using too much antibiotics in animals can make it more difficult to treat some diseases in humans (n=302)	True	54.3 (164)
	False	32.1 (97)
	Cannot answer	13.6 (41)
Antibiotic resistance in human bacteria is only linked to the use of antibiotics in humans and not in animals (n=299)	True	62.9 (188)
	False	24.1 (72)
	Cannot answer	13.0 (39)

Table S9. Discrimination and difficulty values with 95% CIs for items in the three scales (Practices 1, Practices 2 and Knowledge) based on responses in a questionnaire distributed among small- and medium-scale chicken farmers in Thai Nguyen City, Dong Hy and Vo Nhai districts in Vietnam. Practices 1 = Ability to perform desirable practices related to disease management and treatment with antibiotics, Practices 2 = Ability to perform desirable practices regarding disease prevention, Knowledge = Knowledge about antimicrobial resistance (AMR) development and spread.

Item	Discrimination (95% CI)	Difficulty θ (95% CI)
Ability to perform desirable practices related to disease management and treatment with antibiotics (Practices 1 scale)		

90. When you use antibiotics to treat disease among chickens/hens, who usually administers the drug?	0.86 (0.50-1.22)	0.81 (0.41-1.22)
94. If the antibiotic treatment of sick chickens/hens is not effective or does not work, what do you usually do?	1.21 (0.74-1.68)	1.21 (0.80-1.62)
88. When you use antibiotics to treat disease among your chickens/hens, for how long do you usually treat them?	1.32 (0.86-1.77)	0.81 (0.52-1.10)
58. If you have access to buying pharmaceuticals/veterinary drugs, where do you most commonly buy them for your chickens/hens?	2.34 (1.29-3.39)	1.91 (1.47-2.35)
84. What do you usually do first when the chickens/hens at your farm get sick?	2.44 (1.59-3.29)	0.26 (0.10-0.43)
83. Who will usually diagnose disease among the chickens/hens at the farm?	4.19 (2.37-6.01)	1.19 (0.99-1.39)
87. From where do you usually get advice on when to use antibiotics for your chickens/hens?	6.32 (2.57-10.07)	0.79 (0.64-0.93)
89. When treating your chickens/hens with antibiotics, whose instructions do you usually follow on how to use them (dose, treatment length, administration route etc.)?	7.55 (1.82-13.29)	0.69 (0.56-0.82)
Ability to perform desirable practices regarding disease prevention (Practices 2 scale)		
53. What do you usually do with chickens/hens that die from disease?	0.73 (0.36-1.12)	-2.06 (-3.03- -1.09)
80. Do you have separate footwear (e.g. gum boots) or plastic boot covers that you use only in the areas where your chickens/hens are kept?	0.98 (0.59-1.37)	0.43 (0.13-0.74)
35. Do your chickens/hens mix with animals from outside your own farm?	1.07 (0.54-1.60)	-2.35 (-3.26- -1.44)
33. Do other animals at the farm have access to the areas where your chickens/hens are kept?	1.09 (0.65-1.54)	0.87 (0.51-1.23)
85. Do you usually isolate chickens/hens that become sick from the rest of the poultry in the flock?	1.42 (0.88-1.97)	-1.32 (-1.71- - 0.93)
78a. To prevent your chickens/hens from becoming sick, do you fence them?	1.75 (1.13-2.37)	-0.45 (-0.67- -0.24)
51. How do you usually handle manure from your chickens/hens?	1.75 (1.09-2.41)	0.17 (-0.03-0.37)
78b. To prevent your chickens/hens from becoming sick, do you usually isolate/quarantine newly bought animals for some time before mixing them with the chickens/hens that are already at the farm?	1.88 (1.22-2.53)	-0.24 (-0.44- -0.05)
56. If you do not use an all-in/all-out system, how often do you remove litter, manure and clean/disinfect animal houses/areas where the chickens/hens are kept?	2.02 (1.25-2.80)	-0.61 (-0.84- -0.39)
Knowledge about antimicrobial resistance development and spread (Knowledge scale)		
99g. Using antibiotics too often can make diseases difficult to treat in the future (true/false)	0.68 (0.34-1.01)	-1.48 (-2.19- -0.78)
99k. Resistance against antibiotics can make it more difficult to succeed with antibiotic treatment in animals when they get sick (true/false)	1.46 (0.95-1.96)	-1.20 (-1.52- -0.87)
99l. Bacteria resistant to antibiotics can spread from one animal to another (true/false)	2.06 (1.42-2.71)	-0.72 (-0.92- -0.52)
99p. Using too much antibiotics in animals can make it more difficult to treat some diseases in humans (true/false)	3.34 (2.19-4.49)	-0.20 (-0.33- -0.06)
99o. Bacteria resistant to antibiotics can spread through manure from animals (true/false)	3.85 (2.50-5.20)	-0.14 (-0.27- -0.01)
99m. Bacteria resistant to antibiotics can spread between animals and humans (true/false)	4.19 (2.60-5.78)	-0.13 (-0.25-0.00)
99n. Bacteria resistant to antibiotics can spread from animals to humans through animal source foods, e.g. meat (true/false)	8.47 (1.43-15.52)	0.03 (-0.08-0.14)

Table S10. One-way ANOVA with Bonferroni post-hoc test comparing theta means between groups of small- and medium-scale chicken farmers in Thai Nguyen City, Dong Hy and Vo Nhai districts in Vietnam. 5% significance level. Letters indicate significant differences between groups (for each scale). Practices 1 = Ability to perform desirable practices related to disease management and treatment with antibiotics, Practices 2 = Ability to perform desirable practices regarding disease prevention, Knowledge = Knowledge about antimicrobial resistance (AMR) development and spread.

**Description**	**N (305)**	**Theta mean (SE) Practice 1**	**Theta mean (SE) Practice 2**	**Theta mean (SE) Knowledge**
**Overall**	305	0.005 (0.048)	-0.0002 (0.049)	-0.075 (0.049)
**District**				
Dong Hy	56	-0.126^{a} (0.110)	-0.258^{a} (0.135)	-0.310^{a} (0.134)
Thai Nguyen City	51	0.616^{b} (0.110)	0.258^{b} (0.084)	0.190^{b} (0.099)
Vo Nhai	198	-0.116^{a} (0.056)	0.006^{a,b} (0.060)	-0.078^{a,b} (0.060)
**Respondent's sex**				
Female	118	0.017^{a} (0.081)	0.098^{a} (0.073)	-0.059^{a} (0.077)
Male	187	-0.003^{a} (0.059)	-0.062^{a} (0.064)	-0.086^{a} (0.065)
**Age of respondent**				
21-30	11	0.681^{a} (0.243)	-0.172^{a} (0.266)	-0.146^{a} (0.289)
31-40	53	0.038^{a} (0.120)	-0.191^{a} (0.116)	-0.068^{a} (0.118)
41-50	76	0.007^{a} (0.097)	0.120^{a} (0.106)	-0.046^{a} (0.094)
51-60	105	-0.050^{a} (0.075)	-0.042^{a} (0.079)	-0.082^{a} (0.083)
>60	59	-0.049^{a} (0.112)	0.122^{a} (0.105)	-0.102^{a} (0.125)
**Livestock keeping experience of respondent (years)**				
1-10	68	0.461^{a} (0.106)	-0.071^{a} (0.099)	-0.108^{a} (0.098)
11-20	106	-0.148^{b} (0.078)	-0.054^{a} (0.088)	-0.044^{a} (0.082)
21-30	83	-0.030^{b} (0.087)	0.084^{a} (0.099)	-0.138^{a} (0.093)
31-40	40	-0.259^{b} (0.108)	0.154^{a} (0.100)	0.0085^{a} (0.155)
>40	8	-0.176^{a,b} (0.209)	-0.331^{a} (0.245)	0.012^{a} (0.398)
**Education level of respondent**				
Never went to school	5	-0.339^{a} (0.293)	-1.122^{a} (0.435)	-1.184^{a} (0.172)
Primary school	79	-0.139^{a} (0.087)	-0.264^{a,b} (0.098)	-0.465^{a} (0.104)
Secondary school	131	0.048^{a} (0.075)	0.045^{b,c} (0.071)	0.021^{b} (0.072)
High school	84	0.105^{a} (0.090)	0.203^{c} (0.082)	0.150^{b} (0.077)
College/University	6	-0.156^{a} (0.477)	0.568^{b,c} (0.428)	0.726^{b} (0.278)
**Main reason for keeping chickens/hens**				
Commercial	72	0.198^{a} (0.108)	-0.011^{a} (0.105)	-0.121^{a} (0.096)
Household consumption	220	-0.047^{a} (0.054)	0.014^{a} (0.056)	0.001^{a} (0.058)
Other	13	-0.189^{a} (0.188)	-0.177^{a} (0.293)	-1.115^{b} (0.105)
**Farm size**				
≤90	165	-0.079^{a} (0.062)	-0.173^{a} (0.065)	-0.198^{a} (0.065)
≥100	140	0.104^{a} (0.073)	0.203^{b} (0.070)	0.069^{b} (0.073)
**Access to animal health services**				
Yes	97	0.388^{a} (0.096)	-0.088^{a} (0.093)	-0.246^{a} (0.075)
No	198	-0.161^{b} (0.051)	0.030^{a} (0.059)	-0.0001^{b} (0.065)

2.2 Extended result presentation of the univariable analysis related to Table S10.

The results from the one-way ANOVA conducted after the generation of the three IRT scales (Practices 1, relating to disease management and antibiotic treatment; Practices 2, relating to disease prevention; and Knowledge) are shown in Table S10. For all three scales, significant differences in ability and knowledge (mean theta values) were found for district. For Practices 1, the mean theta was significantly lower in Dong Hy and Vo Nhai compared to Thai Nguyen City, whereas for Practices 2 and Knowledge, the mean theta was only significantly lower in Dong Hy compared to Thai Nguyen City.

For the Practices 1 scale, additional significant differences in theta means were found for livestock keeping experience and access to animal health services. The group with a livestock experience of 1-10 years had a significantly higher mean theta score compared to the groups of 11-20, 21-30 and 31-40 years. The group of respondents that had access to animal health services had a significantly higher mean theta score compared to the group without access.

Besides district, variables where significant differences were found for the Practices 2 scale were education level of the respondent and farm size. The group that never went to school had a significantly lower mean theta value than respondents that went to secondary school or higher, and the group that went to primary school had a significantly lower mean theta value than the group that went to high school. Regarding farm size, the group with small farms (<100 birds) had a significantly lower mean value than the large farm group.

For the Knowledge scale, variables with significant differences besides district were education level, main reason for keeping chickens/hens, farm size and access to animal health services. The groups that had never went to school or only primary school had significantly lower mean theta values than the groups with higher education. The groups that kept chickens/hens for household consumption or commercial purpose had significantly higher theta means than the group called "Other" (mainly respondents that kept chickens/hens for both purposes). As for the Practices 2 scale, the group of smaller farms had lower mean thetas than the larger farm group. Finally, the group that did have access to animal health services had a lower mean theta value than the group with access.
